# Rare Variants in *APP*, *PSEN1* and *PSEN2* Increase Risk for AD in Late-Onset Alzheimer's Disease Families

**DOI:** 10.1371/journal.pone.0031039

**Published:** 2012-02-01

**Authors:** Carlos Cruchaga, Sumitra Chakraverty, Kevin Mayo, Francesco L. M. Vallania, Robi D. Mitra, Kelley Faber, Jennifer Williamson, Tom Bird, Ramon Diaz-Arrastia, Tatiana M. Foroud, Bradley F. Boeve, Neill R. Graff-Radford, Pamela St. Jean, Michael Lawson, Margaret G. Ehm, Richard Mayeux, Alison M. Goate, for the NIA-LOAD/NCRAD Family Study Consortium

**Affiliations:** 1 Department of Psychiatry and Hope Center Program on Protein Aggregation and Neurodegeneration, Washington University, St. Louis, Missouri, United States of America; 2 Department of Genetics, Washington University, St. Louis, Missouri, United States of America; 3 Department of Medical and Molecular Genetics, Indiana University, Indianapolis, United States of America; 4 Taub Institute for Research on Alzheimer's Disease and the Aging Brain, Columbia University College of Physicians and Surgeons, New York, New York, United States of America; 5 VA Medical Center and Departments of Neurology and Medicine, University of Washington, Seattle, Washington, United States of America; 6 Department of Neurology, University of Texas Southwestern Medical Center, Dallas, Texas, United States of America; 7 Department of Neurology, Mayo Clinic, Rochester, Minnesota, United States of America; 8 Department of Neurology, Mayo Clinic, Jacksonville, Florida, United States of America; 9 Genetics, GlaxoSmithKline, Research Triangle Park, North Carolina, United States of America; Oslo University Hospital, Norway

## Abstract

Pathogenic mutations in *APP*, *PSEN1*, *PSEN2*, *MAPT* and *GRN* have previously been linked to familial early onset forms of dementia. Mutation screening in these genes has been performed in either very small series or in single families with late onset AD (LOAD). Similarly, studies in single families have reported mutations in *MAPT* and *GRN* associated with clinical AD but no systematic screen of a large dataset has been performed to determine how frequently this occurs. We report sequence data for 439 probands from late-onset AD families with a history of four or more affected individuals. Sixty sequenced individuals (13.7%) carried a novel or pathogenic mutation. Eight pathogenic variants, (one each in *APP* and *MAPT*, two in *PSEN1* and four in *GRN*) three of which are novel, were found in 14 samples. Thirteen additional variants, present in 23 families, did not segregate with disease, but the frequency of these variants is higher in AD cases than controls, indicating that these variants may also modify risk for disease. The frequency of rare variants in these genes in this series is significantly higher than in the 1,000 genome project (p = 5.09×10^−5^; OR = 2.21; 95%CI = 1.49–3.28) or an unselected population of 12,481 samples (p = 6.82×10^−5^; OR = 2.19; 95%CI = 1.347–3.26). Rare coding variants in *APP*, *PSEN1* and *PSEN2*, increase risk for or cause late onset AD. The presence of variants in these genes in LOAD and early-onset AD demonstrates that factors other than the mutation can impact the age at onset and penetrance of at least some variants associated with AD. *MAPT* and *GRN* mutations can be found in clinical series of AD most likely due to misdiagnosis. This study clearly demonstrates that rare variants in these genes could explain an important proportion of genetic heritability of AD, which is not detected by GWAS.

## Introduction

Mutations in *amyloid-beta precursor protein* (*APP*), *presenilin 1* and 2 (*PSEN1, PSEN2*) are known to cause early onset (<60 years) familial Alzheimer disease (AD) [1]–[5]. While initially considered to solely cause early onset AD (EOAD), mutations in *PSEN1* and *PSEN2* have been reported in several families with both late and early onset disease [6]–[10]. Screening for mutations in *PSEN1* has been performed mainly in early-onset families, although a small number of families including late onset (>60 years) cases (<100 families) were also screened [2], [6]–[8], [11]–[14]. Similarly, mutation screening efforts in *APP*, which have historically been limited to exons 16 and 17, have focused on early onset families but have included some late onset families [11], [15]–[17]. Screening of 40 late-onset AD (LOAD) families for *APP* mutations in the exons 16–17 identified a pathogenic *APP* mutation (Glu693Gly) with incomplete penetrance in a single family [17]. Mutations in *progranulin* (*GRN*) and in *microtubule associated protein tau* (*MAPT*) are an established cause of familial frontotemporal dementia (FTD) [18], [19]. Some individuals carrying these mutations have been reported with clinical manifestations indistinguishable from AD [20]–[28], but the frequency of mutations in *GRN* and *MAPT* in clinical series of LOAD cases is unknown. The identification of mutations in these genes in cases with clinically diagnosed AD would change not only the diagnosis and treatment but create the need for genetic counseling.

While there has been considerable success in the identification of genes contributing to EOAD, LOAD is far more frequent, accounting for 99% of all AD cases. LOAD remains a less well understood disorder. The major genetic risk factor for LOAD is *APOE* genotype [29]. The frequency of mutations in *APP*, *PSEN1*, *PSEN2* in LOAD families is unknown because no study has systematically screened these genes in a large series of LOAD families. Because LOAD is so much more common than EOAD, the presence of even a low frequency of mutations would represent a large number of affected families.

Alzheimer Disease is a complex disease and shows heritability of up to 80% [30]. Most recent genetic studies of AD have focused on the identification of common variants associated with risk for AD through genome-wide association studies (GWAS). These studies have identified several new genes that show significant association after multiple test correction in multiple datasets: *CLU*, *PICALM*, *BIN1*, *CR1*, *ABCA7*, *MS4A6A*, *CD33* and *CD2AP* [31]–[35]. However, each of these new signals only account for about 4–9% of the variance in AD susceptibility [36]. The total proportion of heritability explained by the genes that show an association with AD (including APOE) is estimated to be 23% [37], therefore a large proportion of the heritability for LOAD remains unexplained.

GWAS studies are only able to identify common variants that are associated with disease. Importantly, the effect of rare variants cannot be determined in these studies. It has been suggested that the combined effect of rare deleterious mutations could explain a substantial fraction of genetic susceptibility to many common diseases [38]–[43]. Rare alleles can only be identified through resequencing large populations.

In this study we have screened a large collection of LOAD families for mutations in *APP*, *PSEN1*, *PSEN2*, *MAPT and GRN*. We used a method that combines a pooled-DNA approach with next-generation sequencing technology and bioinformatics analyses [44], [45] to identify rare and novel variants in these genes in 439 families with a history of LOAD and/or dementia in four or more family members (Table 1 and 2). This study allows us to accurately estimate the mutation frequency in these genes in LOAD and the effect of rare variants with risk for disease.

**Table 1:** Distribution of the families by the number of affected individuals in each family.

# affected	# families	%	# sequence variants	%
28	1	0.23	1	1.67
20	2	0.46	2	3.33
19	1	0.23	0	0.00
17	1	0.23	2	3.33
16	1	0.23	0	0.00
15	1	0.23	0	0.00
14	1	0.23	0	0.00
13	5	1.14	1	1.67
12	3	0.68	0	0.00
11	4	0.91	0	0.00
10	13	2.96	2	3.33
9	14	3.19	2	3.33
8	27	6.15	5	8.33
7	32	7.29	6	10.00
6	54	12.30	5	8.33
5	107	24.37	9	15.00
4	172	39.18	25	41.67
TOTAL	439		60	

The number of families with different numbers of affected individuals is shown. More than 50% of the families have 4 or 5 affected individuals. The number of sequence variants found in each group is also shown.

**Table 2:** Demographic data for the cohort.

AAO (years)	Sequenced sample	69.92±8.37(range 30–92)
	Families (mean age at onset)	72.80±5.62(range 60–89)
Ethnicity	European ancestry	76.5%
	White- Hispanics	14.6%
	Black- Hispanics	2.7%
	African-American	2.7%
	others	3.6%
Diagnosis	Definite AD	26.94%
	Probable AD	71.46%
	Possible AD	1.59%

The mean, the standard deviation and the range for the age at onset (AAO) for the sequenced samples and the families are shown. For the families the AAO represents the mean AAO of the affected individuals of each family.

## Results

We identified 33 nonsense, missense and splice-site sequence variants in 60/439 (13.7%) individuals, including five known pathogenic variants in ten individuals (2.3%), and three novel potentially pathogenic alleles in four individuals (0.9%). An additional variant in *GRN*, reported previously to be non-pathogenic, because it was present in a very small number of unaffected samples, segregated perfectly with disease status, was not present in 2,692 control chromosomes and may be pathogenic. The fact that it was present in control individuals in previous studies may be because of incomplete penetrance. Pathogenic or likely-pathogenic variants were found in all genes except *PSEN2* (Table 3). The frequency of rare variants in *APP*, *PSEN1* and *PSEN2* in this dataset was significantly higher than the frequency of rare variants found in these genes in three series not enriched for AD cases (GlaxoSmithKline study (GSK study), 1,000 genome project and Exome Variant Server). In the GlaxoSmithKline study (see Materials and Methods and Supporting Materials and Methods S1; n = 12,481), a total of 376 novel and rare non-synonymous, variants were found in *APP*, *PSEN1* and *PSEN2* (2.91% of the samples), but we found 28 rare non-synonymous variants (6.38%) in our 439 samples (p = 6.82×10^−5^; OR = 2.19; 95%CI = 1.347–3.26). In the 1,000 genomes project only 25 rare (MAF<0.05) non-synonymous, nonsense or splice-site variants were identified in these genes from three different populations (CEU, YRI and CHBJPT; (p = 5.09×10^−5^; OR = 2.21; 95%CI = 1.49–3.28; see Supporting Materials and Methods S1). The Exome Variant Server (http://evs.gs.washington.edu/EVS/) [46], is a database with exome sequencing data for 1,326 European-American and 1,067 African-American selected for heart, lung and blood disorders. In this database there were reported 198 novel and rare non-synonymous, nonsense or splice-site variants in these 2,393 individuals, which is statistically lower than in our series (p = 1.79×10^−4^; OR = 1.79; 95%CI = 1.31–2.43). These results most likely reflect the increased recurrence risk among densely affected LOAD families. It is possible therefore that some of these new variants could be pathogenic or disease modifiers.

**Table 3:** Number of rare non-synonymous and splice-site variants found in the 439 sequenced samples.

	Previously reported						New**							
	Pathogenic*		Path. Unkown*		Not Path.*		Likely Pathogenic*		Path. Unkown*		Not Path.*		total	
	#mut	#fam	#mut	#fam	#mut	#fam	#mut	#fam	#mut	#fam	#mut	#fam	#mut	#fam
APP	0	0	0	0	1	1	1	1	2	2	2	3	6	7
PSEN1	2	7	0	0	0	0	0	0	1	1	0	0	3	8
PSEN2	0	0	3	13	0	0	0	0	0	0	0	0	3	13
MAPT	0	0	1	1	0	0	1	1	1	1	4	12	7	15
GRN	3	3	1	1	4	4	1	2	3	4	2	3	14	17
total	5	10	5	15	5	5	3	4	7	8	8	18	33	60
		2.3%		3.4%		1.1%		0.9%		1.8%		4.1%		13.7%

*Based on our segregation analyses.

**Not present in the AD&FTD mutation database or in dbSNP.

Only the rare (MAF<0.05) non-synonymous, splice-site and nonsense sequence variants are shown.

### Pathogenic or likely pathogenic mutations

We identified eight sequence variants, one each in *APP* and *MAPT*, two in *PSEN1*, and four in *GRN*, in a total of 14 families (3.2% of the total families) that are known to cause disease or appear to be highly penetrant rare pathogenic alleles, based on segregation data, bioinformatics and sequence data in additional controls. Three of these variants are novel and five were previously reported (Table 4, 5 and Table S2). The sequence variants showing perfect segregation were the previously reported pathogenic G206A mutation in *PSEN1* [2], [4], the previously reported pathogenic variant *GRN* R110X [18], [47] and *GRN* G515A. In each family, all of the genotyped affected individuals carried the sequence variants, but none of the unaffected individuals were carriers. *PSEN1* G206A and *GRN* R110X were not found in 1,806 AD cases and 1,346 unrelated controls. *GRN* G515A was found in an additional AD case of European-descent (from the 1,806 screened) but not in the 1,346 unrelated controls.

**Table 4:** List of the pathogenic and likely pathogenic non-synonymous, splice site and nonsense sequence variants identified.

Gene	Change	# of fam	Status	Polyphen2prediction	Affected		Unaffected	
					Carriers	Non-carriers	Carriers	Non-carriers
Pathogenic or likely pathogenic								
APP	N660Y	1	Novel	probably damaging	3	0	1	1
					61.6±3	-	62	69
PSEN1	A79V	4	Previously reported Pathogenic	probably damaging	10	1	3	13
					68.9±8.5	77	60±4.3	70.62±12.95
	G206A	3	Previously reported Pathogenic	probably damaging	6	0	0	1
					60.8±6.7	-	-	40
MAPT	G201S	1	Novel	probably damaging	2	0	2	2
					74±0	-	61±5.7	49±4.2
GRN	R110X	1	Previously reported Pathogenic	probably damaging	3	0	0-	5
					66.3±2.1	-	-	72.2±5.26
	c.1414-1G>T	2	Novel	probably damaging	8	1	0	1
					65.8±11.7	74	-	70
	R493X	1	Previously reported Pathogenic	probably damaging	1	3	4	3
					70	78.3±1.15	56.3±4.04	56.3±11.06
	G515A	1	Previously reported Not pathogenic	probably damaging	3	0	0	1
					80±7.5	-	-	66

List of the non-synonymous, splice and nonsense variants identified in the 439 sequenced samples. The identified variants were genotyped in all the available family samples. The number of affected carriers, non-carriers and the un-affected carriers, non-carriers, as well as the mean age at onset and the standard deviation for the affected and the age at last assessment for the unaffected individuals are shown.

The variants were classified as pathogenic, or likely pathogenic based on our segregation analyses, bioinformatic analyses, sequencing and genotyping data in additional cases and controls (table 5) and previous reports.

**Table 5:** Allele frequency of all variants in the initial dataset and in additional case-control series.

			familial cases		AD cases		Controls+unselected	
Likely pathogenic		Ethnicity	Counts	%	Counts	%	Counts	%
APP	N660Y	European	1/439	0.228	0/1,806	0.000	0/(1,346+12,481)	0.000
MAPT	G201S	European	1/439	0.228	1/1,806	0.055	0/1,346	0.000
GRN	c.1414-1G>T	Hispanics	2/439	0.456	0/1,806	0.000	0/1,346	0.000
	G515A	Hispanics	1/439	0.228	1/1,806	0.055	0/1,346	0.000
Likely non-pathogenic								
APP	G322A	Hispanics	1/439	0.228	0/1,806	0.000	0/1,346	0.000
	E599K	European	2/439	0.456	2/1,806	0.111	2/1,346	0.148
	A673T	European	1/439	0.228	0/1,806	0.000	0/1,346	0.000
PSEN2	R62H	Mixed	6/439	1.367	18/1,806	0.997	13/1,346	0.965
	R71W	European	6/439	1.367	26/1,806	1.439	3/1,346	0.222
	M174V	European	1/439	0.228	3/1,806	0.166	0/1,346	0.000
MAPT	R5H	European	1/439	0.228	1/1,806	0.055	0/1,346	0.000
	R168C	European	1/439	0.228	3/1,806	0.166	0/1,346	0.000
	A152T	European	5/439	1.140	17/1,806	0.941	6/1,346	0.445
	V224G	European	2/439	0.456	10/1806	0.553	12/1,346	0.891
	A239T	European	4/439	0.911	4/1,806	0.221	4/1,346	0.297
GRN	P85A	European	2/439	0.456	0/1,806	0.000	0/1,346	0.000
	V141I	European	1/439	0.228	0/1,806	0.000	0/1,346	0.000
	T268M	European	1/439	0.228	1/1,806	0.055	0/1,346	0.000
	A324T	European	1/439	0.228	5/1,806	0.277	2/1,346	0.148
	D376N	European	1/439	0.228	0/1,806	0.000	0/1,346	0.000
	R433Q	European	1/439	0.228	0/1,806	0.000	0/1,346	0.000
Unknown								
APP	G191E	European	1/439	0.228	0/1,806	0.000	0/(1,346+12,481)	0.000
	V340M	Hispanics	1/439	0.228	0/1,806	0.000	0/(1,346+12,481)	0.000
PSEN1	P7L	European	1/439	0.228	0/1,806	0.000	0/1,346	0.000
MAPT	S427F	Hispanics	1/439	0.228	0/1,806	0.000	0/1,346	0.000
GRN	D135V	Hispanics	1/439	0.228	0/1,806	0.000	0/1,346	0.000
	M207T	European	1/439	0.228	0/1,806	0.000	0/1,346	0.000
	V514M	European	1/439	0.228	0/1,806	0.000	0/1,346	0.000
	V519M	Hispanics	2/439	0.456	2/1,806	0.110	7/1,346	0.520

The novel variants that were classified as likely pathogenic or unknown were genotyped in an additional 1,806 unrelated AD cases and 1,346 controls. Among the 1,346 controls 113 are of Hispanic origin. The GSK dataset (n = 12,481, were also used to analyze the presence of some variants in *APP*, *PSEN1* and *PSEN2*).

**Figure 1:**
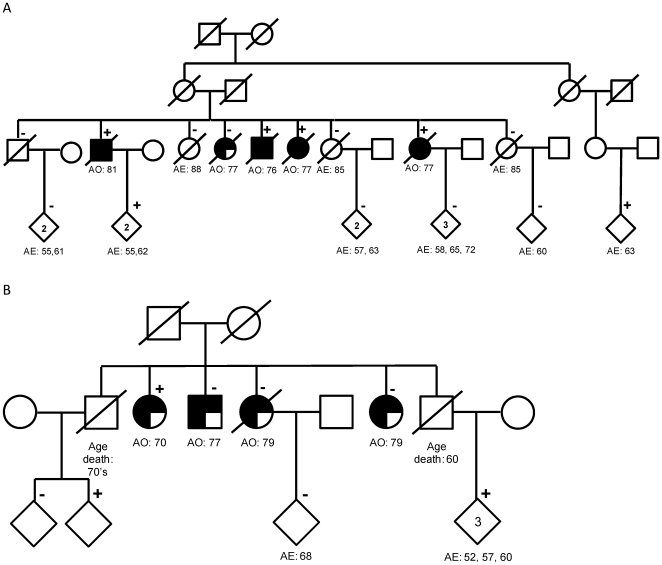
Pedigrees for some of the sequenced families illustrating the presence of phenocopies and low penetrance mutations or the presence of presymptomatic cases. A) Pedigree for a family with the *PSEN1 A79V* mutation. B) Pedigree for a family with the *GRN R493X* mutation. AO indicates the subject or family report of age of onset of symptoms. AE = the age of last evaluation. MCI: Mild cognitive impairment or questionable dementia by family report.+symbol indicates that the subject is positive for the indicated mutation.−symbol indicates that the subject is negative for the indicated mutation. A number inside of a diamond indicates the number of subjects with the same status. Fully shaded circles or squares indicate confirmed AD by autopsy. Three/fourths shaded symbol indicates probable AD diagnosed using NINCDS-ADRDA criteria. One/fourth shaded symbol indicates that the family reports this individual has AD.

The *PSEN1* A79V mutation was found in four of the 439 sequenced samples (Table 3 and 4), including the most densely affected family with 28 affected individuals (Table 1). The mutation in this family was reported previously by sequencing samples with extreme cerebrospinal fluid Aβ levels [6]. The sequenced individual from this family had autopsy confirmed AD and an AAO of 76 years, which was similar to the mean AAO in the entire family. Although A79V is a known pathogenic mutation, seen in families with early onset AD [1], [2], the mutation did not show perfect segregation with disease in this family (Figure 1A). One of the four genotyped affected individuals (AAO 77 years) did not carry the mutation, possibly representing a phenocopy. In addition, three unaffected individuals carried the mutation but their mean age at last assessment was more than ten years below the mean onset of disease in the family. These individuals are likely presymptomatic. *PSEN1* A79V was found also in a sporadic AD case (from the 1,806 screened), but not in the 1,346 unrelated controls.

For the novel variants *APP* N660Y (exon 16) and *MAPT* G201S (exon 9), some of the carriers were asymptomatic at the time of the last assessment (Table 4), but in all cases, the age at last assessment of the unaffected carriers is lower than the oldest age at onset for the affected carriers. Both *APP* N660Y, and *MAPT* G201S are predicted to be damaging by Polyphen2 [48]. The *APP* N660Y variant was not found in 1,806 AD cases, 1,346 unrelated controls, nor in the 12,481 subjects not enriched for AD (GSK study), nor in the Exome Variant Server. The *MAPT* G201S variant was found in an additional case from the NIA-LOAD study (from the 847 screened). The *MAPT* G201S variant was present in the affected sibling but not in an unaffected family member. This variant was not found in 1,346 unrelated elderly non-demented controls (Table 5), nor in the Exome Variant Server.

An additional known pathogenic mutation, R493X, and a novel potentially pathogenic variant, c.1414-1G>T, were identified in *GRN*. However as with the *PSEN1* A79V mutation, these *GRN* variants do not show perfect segregation (Table 4, Figure 1B). The R493X mutation was present in a single demented individual (AAO = 70), but was absent in another three demented individuals (AAO = 78.3±1.15), suggesting that these non-carriers are phenocopies. Four young unaffected individuals are also mutation carriers. The R493X has been found in more than 12 FTD kindreds (AD&FTD mutation database) and functional data has shown that the mutation results in a premature termination codon causing nonsense-mediated mRNA decay [18], [19]. In the families with the *GRN* c.1414-1G>T variant we also found a single affected individual who did not carry the sequence variant. The age at onset of this individual is higher than the mean onset for the affected individuals carrying the sequence variant, suggesting that this individual may be a phenocopy. The *GRN* splice-site variant c.1414-1G>T affects the last nucleotide of intron 10 within a core splice-site. This variant was found in two Hispanic families, one of which had 20 affected individuals. This variant is predicted to alter an acceptor splice-site (NetGene2 Prediction score for the wild type variant 1.00 vs. 0.00 for the mutant) causing exon skipping. A recent screen for *GRN* pathogenic mutations identified a similar variant (c.1414-2A>G) affecting the same core splice-site. *Ex vivo* splicing assays confirmed that the mutation c.1414-2A>G affects splicing of the exon [47]. Neither the *GRN* R493X nor the c.1414-1G>T variant were found in the controls or in the sporadic AD cases (Table 5), nor the Exome Variant Server. None of the sequenced individuals with pathogenic or likely pathogenic mutations in *GRN* or *MAPT* had neuropathologic confirmation of diagnosis. These individuals most likely have frontotemporal dementia that has a clinical presentation indistinguishable from AD.

The literature [47], segregation data, the absence of variant carriers among non-demented controls, and our bioinformatic analyses strongly suggest that *APP* N660Y, *MAPT* G201S and *GRN* c.1414-1G>T are rare pathogenic alleles exhibiting high disease penetrance.

### Non-pathogenic or likely non-pathogenic variants

We identified a total of thirteen (eight novel) sequence variants (23 families) which clearly did not segregate with disease and/or were found in controls suggesting that these variants are not causative mutations (Table 5 and Table S2), but our analyses suggest that some of them may be disease modifiers. Some of these variants (*PSEN2* R71W and M174V, and *MAPT* R5H) were previously classified as pathogenic in the AD&FTD mutation database; however our results do not support this designation. These variants occurred in multiple families but did not segregate with disease status in any family (Table S2) and were observed multiple times in the unselected samples and the elderly non-demented controls samples (Table 5).

The majority of the genetic variants identified in these genes, so far, have been classified as pathogenic or non-pathogenic, but rare variants in these genes could also be risk factors for disease. Carriers of the R62H and R71W *PSEN2* variants have a significantly earlier age at onset than affected non-carriers even after correcting for *APOE* genotype (R71W: 70.2 vs 76.7, p = 0.0005; R62H: 71 vs 75 years, p = 0.0019; Table S3), suggesting that these variants could be disease modifiers.

The novel variant *APP* G322A was present in four affected individuals but absent in 7 additional affected members from the same family and therefore does not segregate with disease. This variant was not found in 12,481 samples unselected for AD (P = 1.0×10^−7^). The bioinformatic analyses predict it as probably damaging. These data suggest that the *APP* G322A variant may be a risk factor for AD, but more studies will be necessary to confirm this hypothesis.

The frequency of these variants is, in most cases, higher in AD cases than in controls. As these variants have very low frequency, the power to identify significant differences in cases vs. controls is very limited. However, if we combine all of the novel variants classified as non-pathogenic (Table 5), the frequency of these variants in AD cases is significantly higher than in controls (5.66% in AD cases vs 2.89% in controls; p = 3.11×10^−4^; OR = 1.86; 95%CI = 1.30–2.65), suggesting that these variants may increase risk for AD, although they are not causative. See Supporting Results S1 for full results about the likely non-pathogenic variants unknown pathogenicity.

### Variants with unknown pathogenicity

For eight novel variants (*APP* G191E and V340M; *PSEN1* P7L; *GRN* D135V, M207T, V514M, V519M; and *MAPT* S427F), segregation within families was inconclusive, in part due to the small number of family members sampled. The *APP* and *PSEN1* variants were absent from the 12,481 unselected samples, and the *GRN* D135V and V514M and *MAPT* mutation were not found in 1,346 non-demented elderly controls (Table 5). Bioinformatics analyses predict that the *APP*, *MAPT*, *and GRN* V519M variants are probably damaging (Table S2). The total number of variants in AD cases is significantly higher than in controls (2.23% in AD cases vs 0.52% in controls; p = 9.46×10^−12^; OR = 4.38; 95%CI = 1.65–11.57) strongly suggesting that some of these novel variants may cause or increase risk for AD.

### Impact of *APOE* genotype in these families

*APOE* is the strongest known genetic risk factor for sporadic and familial LOAD. Therefore we analyzed the effect of *APOE* in these families. We initially compared the *APOE4* allele frequency in the sequenced samples comparing carriers for rare sequence variants against the non-carriers. There was no significant difference between these two groups (71.15% vs 72.29% p = 0.86). However we found that the *APOE4* allele frequency in these families is significantly higher compared to sporadic AD cases (72.13% vs. 60%, p = 2.18×10^−6^, OR = 1.71, 95%CI: 1.36–2.17) and controls (72.13% vs. 26%, p = 5.28×10^−85^; OR = 7.37, 95%CI: 5.86–9.27)(APOE4 frequencies obtained from Alzgene). These results confirm that *APOE* is a strong genetic risk factor in these families, and suggest that the *APOE4* genotype could be sufficient to cause disease in some of these families, as suggested in a recent report [49]. However, we found likely causative variants in individuals with every *APOE* genotype and no difference in the *APOE4* frequency among the samples with and without rare sequence variants. As reported previously, we found that *APOE4* is associated with a dose dependent decrease in the AAO (Table S3 and Figure S1). *APOE4* carriers with a rare sequence variant in the screened genes have a lower AAO than *APOE4* carriers without a rare sequence variant in these genes (67.3 y vs. 69.8 y; p = 0.025), suggesting that these sequence variants modify AAO of AD independently of *APOE* genotype.

### Comparison of the individuals and families with and without sequence variants

The age at onset of individuals with rare non-synonymous variants in these genes was significantly lower than that for sequenced individuals with normal gene sequence (67.8 years vs 70.5 years p = 0.004), but there was no difference in the mean age at onset in families with rare non-synonymous variants compared to those without sequenced variants (Table S4). The major feature differentiating the families with novel rare variants from those with normal gene sequence was the reported number of affected individuals per family. We found that the families with rare sequence variants had significantly more affected individuals than families without sequence variants (average = 6.88 vs 5.62; p = 0.0008; Figures S2, S3, S4, Table S4).

## Discussion

Pathogenic mutations in *APP*, *PSEN1*, *PSEN2*, *MAPT* and *GRN* have previously been linked to familial early onset forms of dementia. A recent report looking at common variants in *APP*, *PSEN1*, *PSEN2* and *MAPT* in a large case-control sample consisting of 3,940 cases and 13,373 controls, found that common variants in these genes are unlikely to make strong contributions to susceptibility for LOAD [50]. However, the impact of rare variants in these genes in late onset clinically diagnosed AD remains unclear. This is the first study to systematically screen for rare variants and pathogenic mutations in *APP*, *PSEN1*, *PSEN2*, *MAPT* and *GRN* in a large clinical series of well-characterized families densely affected by LOAD.

We found seven families carrying two known causative mutations in *PSEN1* (A79V and G206A [1]–[4]), and three families carrying two clearly causative mutations in *GRN* (R110X, R493X [19], [22], [47], [51]–[54]. In some families these causative mutations (*PSEN1*-A79V, and *GRN*-R493X, Figure 1) did not completely segregate with disease, illustrating how phenocopies, potential presymptomatic individuals and reduced penetrance may complicate the interpretation of novel sequence variants in familial and sporadic LOAD. While we can identify the phenocopies in families with known mutations, as in the case of *PSEN1* A79V and *GRN* R110X (Fig. 1), it is more challenging in families with novel variants, because phenocopies might be interpreted as a failure of the variant to segregate with disease. Putative pathogenic variants in genes that cause late onset rather than early onset dementia could have a less severe effect on protein function due to other genetic or environmental modifiers that are not present when these same variants result in early onset disease. This would lead to the occurrence of reduced penetrance. Indeed, several cases of reduced penetrance have been reported in families with *PSEN1* and *GRN* mutations [51], [55]–[58]. Despite these problems in our segregation data, the absence of these variants in a large non-demented control series and the bioinformatics analyses suggest that the novel variants *APP* N660Y, *GRN* c.1414-1G>T and *MAPT* G201S are likely to be causative mutations or highly penetrant rare disease alleles.

In total 14 (3.2%) of the 439 sequenced samples from densely affected families carried a causative or likely-causative mutation. Although this percentage is low, the overall number of LOAD cases carrying mutations in these genes is likely to be higher than the number of early onset cases, because LOAD is much more frequent. Approximately 10–20% of all patients with LOAD have a family history of dementia. Of those, approximately 10% have a history of four or more affected family members. Based on our analysis, we estimate 3.2% of these densely affected families would have a causative mutation. Thus, 0.029–0.057% of all patients with LOAD may carry a pathogenic mutation in these genes, representing a minimum of 1,917–3,833 cases in the United States.

It is likely that we have underestimated the actual number of cases with causative mutations. First, the recurrence risk in family members peaks with an age-at-onset of 85 years in the proband [59], [60]. Several families with more than 10 affected individuals have no mutations in the genes studied here. Thus it is very likely that there are novel AD causative genes yet to be discovered. Second, some of the variants with unknown pathogenicity reported in this study, such as, *APP G220E* and V340M, *and MAPT* S427F, could be pathogenic, or risk factors. The scarcity of available DNA samples from other family members with LOAD made it difficult to determine whether or not these novel variants segregate with disease. However, we did not find these variants in additional controls and the bioinformatic analyses suggest that these variants may be pathogenic. To confirm this hypothesis more genetic and functional analyses will be necessary. Lastly, some of the variants we found in this study (*APP* A79V, *MAPT* G201V and *GRN* G515A) were also found in sporadic cases, but not in controls. These results indicate that novel mutations remain to be discovered and that mutations in these genes are also present in LOAD cases with no clear family history. Together these results suggest that any individual with a family history of dementia affecting a large number of relatives and with an onset in the mid-60s should be considered for mutation screening not only of the AD genes (*APP*, *PSEN1* and 2), but also *MAPT* and *GRN*. Our study also indicates that mutations in these genes can be present in 1 to 3% of the sporadic cases. These cases may be classified as sporadic because the family size is small, because of the lack of medical records for other family members or because the variant has low penetrance.

Another important finding is the observation of clinically diagnosed AD families carrying previously reported and likely novel pathogenic mutations in *GRN* and *MAPT*. Mutations in these genes are typically associated with frontotemporal lobar degeneration [18], [19], but have been previously reported in clinically diagnosed AD cases [20]–[28]. Our results show that mutations in *GRN* and *MAPT* in a clinical series of LOAD families are as common as mutations in the AD genes (*APP*, *PSEN1* and *2*). Six families (1.37%) with pathogenic or likely pathogenic mutations in *GRN* and *MAPT* were found vs. eight families (1.82%) with mutations in *APP* and *PSEN1*. None of the individuals with *MAPT* or *GRN* mutations had autopsy confirmation of the clinical diagnosis. It is most likely that these individuals have been misdiagnosed and that neuropathological diagnosis for these individuals will be FTD and not AD.

Our study indicates that families carrying mutations in *GRN* or *MAPT* can present with disease that is clinically indistinguishable from probable AD even in specialist memory disorder clinics, which is analogous to the observations that “AD mutations” can present with frontotemporal lobar degeneration [61]–[63]. Identification of families and individuals carrying mutations in genes associated with frontotemporal lobar degeneration will be important for clinical management of these patients, particularly as therapies are developed that target the specific pathophysiologic processes of these disorders.

Some of the rare variants discovered in this study appear to increase the risk for AD or modify the age at onset. We found a very significant association of *PSEN2* R62H and R71W with age at onset. It is very likely that some of the variants classified as non-pathogenic also increase risk for AD: When the individuals with variants classified as pathogenic are removed (n = 14), and the frequency of rare non-synonymous variants is compared with 1,000 genome project data (p = 1.93×10^−3^; OR = 2.18, 95%CI: 1.31–3.62), the GSK dataset (p = 5.99×10^−3^; OR = 1.89, 95%CI: 1.19–3.00) or the Exome Variant Server (p = 0.010; OR = 1.48; 95%CI = 1.06–2.06), there is still a significant excess of rare variants in these genes in the LOAD cases. In the three datasets (GSK 1,000 genome project, and Exome Variant Server) a large proportion of samples are of non-European origin, therefore some of the variants found in these datasets may represent population specific alleles, decreasing the power of our analyses. If we focus on the specific variants found in this study and compare their frequency in elderly non-demented individuals, we found that these variants may have a big impact on AD risk. The eight variants classified as “unknown pathogenicity” have a combined OR = 4.38 (95%CI = 1.65–11.57), and the variants classified as “non-pathogenic” have a combined OR = 1.86 (95%CI = 1.30–2.65). The common variants identified in the last GWAS for AD had ORs ranging from 1.26 to 1.11, with a combined OR of 2.23. Therefore an individual carrying a single rare variant in one of these genes has a higher likelihood of developing AD than individuals varying all of the risk alleles for the novel genes found in the recent GWAS studies. This is the first large study looking at the effect of rare variants in candidate genes in AD. Additional large studies are needed to replicate these findings.

This work provides some general guidelines to identify individuals and families that should be prioritized for genetic counseling and mutation screening. Although we did not find clear phenotypic differences between the families carrying missense, splice-site or nonsense variants compared with the families without novel variants, the number of reported affected individuals and the presence of at least one affected individual with an early age at onset were strong indicators of the presence of a pathogenic variant. Given the significant overlap in clinical presentation, individuals with a strong family history of AD should also be screened for mutations in *MAPT* and *GRN*, genes typically associated with frontotemporal lobar degeneration, when no autopsy is available. In families with a history of four or more affected individuals and with an onset in the mid-60s, genetic testing may be considered for *PSEN1-2*, *APP*, *GRN* and *MAPT*. Families with multiplex late onset dementia should be referred to genetic counsellors.

Rare coding variants in *APP*, *PSEN1 and PSEN2*, increase risk for or cause late onset AD. Dividing AD into late onset and early onset is probably not useful from a mechanistic point of view because mutations in *APP*, *PSEN1 and PSEN2* can be found in early onset and late onset AD. Similarly, *APOE4* increases risk for AD in both early and late onset AD. Clearly factors other than the mutation can impact the age at onset and penetrance of at least some variants causing AD. *MAPT* and *GRN* mutations can be found in clinical series of AD most likely due to misdiagnosis. Finding a mutation in these genes would change the clinical diagnosis in a demented individual. This will be particularly important when mechanism-based therapies become available because this would change the treatment of these individuals. Lastly, familial aggregation is more important than age at onset in determining the likelihood of an individual carrying a disease-causing variant.

## Materials and Methods

### Patients

The NIA-LOAD Family Study recruited 992 multiplex LOAD families, with at least two living affected individuals, from throughout the United States. A description of these samples has been reported previously [64]. We selected for sequencing all families (439) that met the following criteria: at least 4 family members reported with a history of dementia consistent with LOAD, and either at least two affected family members reporting an age of onset of 65 years or older or an average age of onset in the family of 60 years or older (Table 1). The youngest affected family member with the most definitive diagnosis was selected for sequencing. In some families the age at onset for the sequenced sample was lower than 60 years, but the mean age at onset for the entire family was equal to or greater than 60 years (Table 2). We chose individuals with autopsy confirmed disease (26.9%) over those with probable (71.4%) or possible (1.5%) disease (NINCDS-ADRDA) [65]. Written consent was obtained from all participants, and the study was approved by the local IRB committees.

### DNA sequencing and genotyping

We used the next-generation, pooled-DNA method described by Druley et al. [44], and Vallania et al. [45] to identify sequence variants in *APP*, *PSEN1*, *PSEN2*, *MAPT* and *GRN*. A more detailed description of this method is provided in the Supporting Materials and Methods S1 and Table S1. All rare (minor allele frequency<5%) missense, nonsense, and splice-site sequence variants identified in the pooled DNA experiment were confirmed and the specific sample carrying the variant was identified by direct genotyping using standard procedures for Sequenom, Taqman or Kaspar. We genotyped the confirmed variants in all available family members to determine whether the sequence variant segregated with disease. Common variants and synonymous variants were not followed up.

### Bioinformatics

PolyPhen2 [48] and Net2Gene [66] were used to evaluate the effect of non-synonymous and splice-site variants on protein function and structure. The AD&FTD mutation database (http://www.molgen.ua.ac.be/ADMutations/) was used to identify sequence variants found in previous studies of early onset familial dementia and to determine whether or not they were considered to be disease-causative variants. The sequencing data from the 1,000 genome project and the GlaxoSmithKline study (GSK study; see below and supporting Materials and Methods S1 and Table S5) were used to estimate the frequency of novel and rare (minor allele frequency less than 5%) missense, nonsense and splice site variants in samples unselected for studies of AD. For *APP*, *PSEN1* and *PSEN2* we had access to sequence data from the GSK study for the exons of these genes including variant counts and allele frequencies for 12,481 individuals from 10 disease collections and 2 population-based studies all unselected for AD. Average age of exam for this sample was 51.7 years. This dataset included 10,967 Caucasians, 594 African-Americans, 566 South Asians, 84 Ashkenazi Jews, 29 Hispanics and 241 individuals of mixed ethnicity (see supporting Materials and Methods S1).

### Genotyping additional cases and controls

All variants classified as “likely pathogenic” or with an “unknown pathogenicity”, and some “non-pathogenic” variants were genotyped in 961 sporadic AD cases, 1,346 unrelated elderly non-demented controls [34], [35] and a single case from each of the remaining NIA-LOAD families (n = 847 with </ = 3 affected individuals) [64].

## Supporting Information

Materials and Methods S1 Additional material and methods.
(DOC)

Results S1 Additional results.
(DOC)

Table S1 Sensitivity and specificity of the next-gen DNA approach.
(DOC)

Table S2 List of all non-pathogenic and unknown pathogenicity rare non-synonymous, splice site and nonsense sequence variants identified.
(DOC)

Table S3 Association of APOE with AAO.
(DOC)

Table S4 Comparison of the sequenced samples and families with and without variants.
(DOC)

Table S5 Summary of sample characteristics for the GSK study.
(DOC)

Figure S1 The number of *APOE 4* alleles is associated with age at onset. **A**) Age at onset was analyzed for association with the number of *APOE 4* alleles in all the affected family members using the Kaplan-Meier method and tested for significant differences, using a proportional hazards model (proc PHREG, SAS). Family and gender were included in the model to take into account the relatedness between samples. Carriers of *APOE 4* alleles have an earlier AAO than non-carriers **B**) Age at onset was analyzed for association with the number of *APOE 4* alleles in the sequenced samples using the Kaplan-Meier method and tested for significant differences, using a proportional hazards model (proc PHREG, SAS). Carriers of *APOE 4* alleles have an earlier AAO than non-carriers.
(DOC)

Figure S2 Number of affected individuals in the families with any sequence variant compared to the families with no sequence variants. The families for which the selected sample carried a sequence variant have a higher mean number of affected individuals (6.88±4.5 (4–28)), than the families without sequence variants (5.62±2.21 (4–19)). For this analysis we included all of the sequence variants identified in this study, even if they were considered non-pathogenic.
(DOC)

Figure S3 The number of affected individuals and the age at onset of the sequenced individual, but not gender or *APOE* genotype are associated with the presence of non-synonymous, nonsense or splice-site variants in the *APP*, *PSEN1*, *PSEN2*, *MAPT* or *GRN* genes. We used a logistic regression model to analyze the association of the number of affected individuals, the age at onset of the sequenced individual, gender and *APOE* genotype with the presence of sequence variants. The Odds Ratio (OR) and the 95% confidence interval were calculated. The risk of having a sequence variant in these genes increase by 1.33 (95%: 1.105–1.69) for every two affected individuals in the family. The risk of having a sequence variant in these genes decreased by 0.807 (95%: 0.67–0.96) for every five years increase in age at onset.
(DOC)

Figure S4 ROC curve for the logistic regression model including the number of affected individuals in a family and age at onset for the presence or absence of sequence variations in the *APP*, *PSEN1*, *PSEN2*, *MAPT* or *GRN* genes. We used a logistic regression model to generate a ROC curve including the variables that could predict the presence of a sequence variant in this series. A stepwise regression analyses was used to include the most significant variables among: *APOE* genotype, gender, age at onset and the number of affected individuals in each family. The logistic regression identified age at onset (p = 0.0008; Area under the Curve = 0.6022) in the first step and number of affected individuals (p = 0.0001; Area under the Curve = 0.5779) in the second step. No other variable entered in the model.
(DOC)

## References

[1] Cruts M, van Duijn CM, Backhovens H, Van den Broeck M, Wehnert A (1998). Estimation of the genetic contribution of presenilin-1 and -2 mutations in a population-based study of presenile Alzheimer disease.. Hum Mol Genet.

[2] Rogaeva EA, Fafel KC, Song YQ, Medeiros H, Sato C (2001). Screening for PS1 mutations in a referral-based series of AD cases: 21 novel mutations.. Neurology.

[3] Brickell KL, Leverenz JB, Steinbart EJ, Rumbaugh M, Schellenberg GD (2007). Clinicopathological concordance and discordance in three monozygotic twin pairs with familial Alzheimer's disease.. J Neurol Neurosurg Psychiatry.

[4] Athan ES, Williamson J, Ciappa A, Santana V, Romas SN (2001). A founder mutation in presenilin 1 causing early-onset Alzheimer disease in unrelated Caribbean Hispanic families.. Jama.

[5] Goate A, Chartier-Harlin MC, Mullan M, Brown J, Crawford F (1991). Segregation of a missense mutation in the amyloid precursor protein gene with familial Alzheimer's disease.. Nature.

[6] Kauwe JS, Jacquart S, Chakraverty S, Wang J, Mayo K (2007). Extreme cerebrospinal fluid amyloid beta levels identify family with late-onset Alzheimer's disease presenilin 1 mutation.. Ann Neurol.

[7] Devi G, Fotiou A, Jyrinji D, Tycko B, DeArmand S (2000). Novel presenilin 1 mutations associated with early onset of dementia in a family with both early-onset and late-onset Alzheimer disease.. Archives of Neurology.

[8] Larner AJ, Ray PS, Doran M (2007). The R269H mutation in presenilin-1 presenting as late-onset autosomal dominant Alzheimer's disease.. J Neurol Sci.

[9] Jayadev S, Leverenz JB, Steinbart E, Stahl J, Klunk W (2010). Alzheimer's disease phenotypes and genotypes associated with mutations in presenilin 2.. Brain: a journal of neurology.

[10] Tomaino C, Bernardi L, Anfossi M, Costanzo A, Ferrise F (2007). Presenilin 2 Ser130Leu mutation in a case of late-onset “sporadic” Alzheimer's disease.. Journal of Neurology.

[11] Arango D, Cruts M, Torres O, Backhovens H, Serrano ML (2001). Systematic genetic study of Alzheimer disease in Latin America: mutation frequencies of the amyloid beta precursor protein and presenilin genes in Colombia.. American journal of medical genetics.

[12] Scacchi R, Gambina G, Moretto G, Corbo RM (2007). A mutation screening by DHPLC of PSEN1 and APP genes reveals no significant variation associated with the sporadic late-onset form of Alzheimer's disease.. Neuroscience letters.

[13] Taddei K, Fisher C, Laws SM, Martins G, Paton A (2002). Association between presenilin-1 Glu318Gly mutation and familial Alzheimer's disease in the Australian population.. Molecular Psychiatry.

[14] de Silva R, Weiler M, Morris HR, Martin ER, Wood NW (2001). Strong association of a novel Tau promoter haplotype in progressive supranuclear palsy.. Neurosci Lett.

[15] Houlden H, Crawford F, Rossor M, Mullan M (1993). Screening for the APP codon 670/671 mutations in Alzheimer's disease.. Neuroscience letters.

[16] Tanzi RE, Vaula G, Romano DM, Mortilla M, Huang TL (1992). Assessment of amyloid beta-protein precursor gene mutations in a large set of familial and sporadic Alzheimer disease cases.. American journal of human genetics.

[17] Kamino K, Orr HT, Payami H, Wijsman EM, Alonso ME (1992). Linkage and mutational analysis of familial Alzheimer disease kindreds for the APP gene region.. American journal of human genetics.

[18] Van Deerlin VM, Wood EM, Moore P, Yuan W, Forman MS (2007). Clinical, genetic, and pathologic characteristics of patients with frontotemporal dementia and progranulin mutations.. Arch Neurol.

[19] Huey ED, Grafman J, Wassermann EM, Pietrini P, Tierney MC (2006). Characteristics of frontotemporal dementia patients with a Progranulin mutation.. Ann Neurol.

[20] Cortini F, Fenoglio C, Guidi I, Venturelli E, Pomati S (2008). Novel exon 1 progranulin gene variant in Alzheimer's disease.. Eur J Neurol.

[21] Kelley BJ, Haidar W, Boeve BF, Baker M, Shiung M (2010). Alzheimer disease-like phenotype associated with the c.154delA mutation in progranulin.. Arch Neurol.

[22] Rademakers R, Baker M, Gass J, Adamson J, Huey ED (2007). Phenotypic variability associated with progranulin haploinsufficiency in patients with the common 1477C−>T (Arg493X) mutation: an international initiative.. Lancet Neurol.

[23] Brouwers N, Nuytemans K, van der Zee J, Gijselinck I, Engelborghs S (2007). Alzheimer and Parkinson diagnoses in progranulin null mutation carriers in an extended founder family.. Arch Neurol.

[24] Lindquist SG, Holm IE, Schwartz M, Law I, Stokholm J (2008). Alzheimer disease-like clinical phenotype in a family with FTDP-17 caused by a MAPT R406W mutation.. European journal of neurology: the official journal of the European Federation of Neurological Societies.

[25] Rademakers R, Dermaut B, Peeters K, Cruts M, Heutink P (2003). Tau (MAPT) mutation Arg406Trp presenting clinically with Alzheimer disease does not share a common founder in Western Europe.. Human mutation.

[26] Momeni P, Pittman A, Lashley T, Vandrovcova J, Malzer E (2009). Clinical and pathological features of an Alzheimer's disease patient with the MAPT Delta K280 mutation.. Neurobiology of aging.

[27] Ludolph AC, Kassubek J, Landwehrmeyer BG, Mandelkow E, Mandelkow EM (2009). Tauopathies with parkinsonism: clinical spectrum, neuropathologic basis, biological markers, and treatment options.. Eur J Neurol.

[28] Reed LA, Grabowski TJ, Schmidt ML, Morris JC, Goate A (1997). Autosomal dominant dementia with widespread neurofibrillary tangles.. Ann Neurol.

[29] The AlzGene Database Alzheimer Research Forum.. http://www.alzgene.org.

[30] Gatz M, Reynolds CA, Fratiglioni L, Johansson B, Mortimer JA (2006). Role of genes and environments for explaining Alzheimer disease.. Archives of general psychiatry.

[31] Harold D, Abraham R, Hollingworth P, Sims R, Gerrish A (2009). Genome-wide association study identifies variants at CLU and PICALM associated with Alzheimer's disease.. Nat Genet.

[32] Lambert JC, Heath S, Even G, Campion D, Sleegers K (2009). Genome-wide association study identifies variants at CLU and CR1 associated with Alzheimer's disease.. Nat Genet.

[33] Seshadri S, Fitzpatrick AL, Ikram MA, DeStefano AL, Gudnason V (2010). Genome-wide analysis of genetic loci associated with Alzheimer disease.. Jama.

[34] Hollingworth P, Harold D, Sims R, Gerrish A, Lambert JC (2011). Common variants at ABCA7, MS4A6A/MS4A4E, EPHA1, CD33 and CD2AP are associated with Alzheimer's disease.. Nature Genetics.

[35] Naj AC, Jun G, Beecham GW, Wang LS, Vardarajan BN (2011). Common variants at MS4A4/MS4A6E, CD2AP, CD33 and EPHA1 are associated with late-onset Alzheimer's disease.. Nature Genetics.

[36] Ertekin-Taner N (2010). Genetics of Alzheimer disease in the pre- and post-GWAS era.. Alzheimers Res Ther.

[37] So HC, Gui AH, Cherny SS, Sham PC (2011). Evaluating the heritability explained by known susceptibility variants: a survey of ten complex diseases.. Genetic epidemiology.

[38] Nejentsev S, Walker N, Riches D, Egholm M, Todd JA (2009). Rare variants of IFIH1, a gene implicated in antiviral responses, protect against type 1 diabetes.. Science.

[39] Azzopardi D, Dallosso AR, Eliason K, Hendrickson BC, Jones N (2008). Multiple rare nonsynonymous variants in the adenomatous polyposis coli gene predispose to colorectal adenomas.. Cancer Res.

[40] Masson E, Chen JM, Scotet V, Le Marechal C, Ferec C (2008). Association of rare chymotrypsinogen C (CTRC) gene variations in patients with idiopathic chronic pancreatitis.. Hum Genet.

[41] Ma X, Liu Y, Gowen BB, Graviss EA, Clark AG (2007). Full-exon resequencing reveals toll-like receptor variants contribute to human susceptibility to tuberculosis disease.. PLoS One.

[42] Kamboh MI, Aston CE, Perez-Tur J, Kokmen E, Ferrell RE (1999). A novel mutation in the apolipoprotein E gene (APOE*4 Pittsburgh) is associated with the risk of late-onset Alzheimer's disease.. Neurosci Lett.

[43] Kim M, Suh J, Romano D, Truong MH, Mullin K (2009). Potential late-onset Alzheimer's disease-associated mutations in the ADAM10 gene attenuate {alpha}-secretase activity.. Hum Mol Genet.

[44] Druley TE, Vallania FL, Wegner DJ, Varley KE, Knowles OL (2009). Quantification of rare allelic variants from pooled genomic DNA.. Nat Methods.

[45] Vallania FL, Druley TE, Ramos E, Wang J, Borecki I (2010). High-throughput discovery of rare insertions and deletions in large cohorts.. Genome Res.

[46] Exome Variant Server.. http://evsgswashingtonedu/EVS/.

[47] Yu CE, Bird TD, Bekris LM, Montine TJ, Leverenz JB (2010). The spectrum of mutations in progranulin: a collaborative study screening 545 cases of neurodegeneration.. Arch Neurol.

[48] Adzhubei IA, Schmidt S, Peshkin L, Ramensky VE, Gerasimova A (2010). A method and server for predicting damaging missense mutations.. Nat Methods.

[49] Genin E, Hannequin D, Wallon D, Sleegers K, Hiltunen M (2011). APOE and Alzheimer disease: a major gene with semi-dominant inheritance.. Molecular Psychiatry.

[50] Gerrish A, Russo G, Richards A, Moskvina V, Ivanov D (2011). The Role of Variation at AbetaPP, PSEN1, PSEN2, and MAPT in Late Onset Alzheimer's Disease.. J Alzheimers Dis.

[51] Gass J, Cannon A, Mackenzie IR, Boeve B, Baker M (2006). Mutations in progranulin are a major cause of ubiquitin-positive frontotemporal lobar degeneration.. Hum Mol Genet.

[52] Pickering-Brown SM, Baker M, Gass J, Boeve BF, Loy CT (2006). Mutations in progranulin explain atypical phenotypes with variants in MAPT.. Brain.

[53] Mesulam M, Johnson N, Krefft TA, Gass JM, Cannon AD (2007). Progranulin mutations in primary progressive aphasia: the PPA1 and PPA3 families.. Arch Neurol.

[54] Spina S, Murrell JR, Huey ED, Wassermann EM, Pietrini P (2007). Clinicopathologic features of frontotemporal dementia with Progranulin sequence variation.. Neurology.

[55] Rossor MN, Fox NC, Beck J, Campbell TC, Collinge J (1996). Incomplete penetrance of familial Alzheimer's disease in a pedigree with a novel presenilin-1 gene mutation.. Lancet.

[56] Llado A, Fortea J, Ojea T, Bosch B, Sanz P (2010). A novel PSEN1 mutation (K239N) associated with Alzheimer's disease with wide range age of onset and slow progression.. Eur J Neurol.

[57] Le Ber I, van der Zee J, Hannequin D, Gijselinck I, Campion D (2007). Progranulin null mutations in both sporadic and familial frontotemporal dementia.. Hum Mutat.

[58] Foster NL, Heidebrink JL, Clark CM, Jagust WJ, Arnold SE (2007). FDG-PET improves accuracy in distinguishing frontotemporal dementia and Alzheimer's disease.. Brain.

[59] Silverman JM, Smith CJ, Marin DB, Mohs RC, Propper CB (2003). Familial patterns of risk in very late-onset Alzheimer disease.. Arch Gen Psychiatry.

[60] Silverman JM, Ciresi G, Smith CJ, Marin DB, Schnaider-Beeri M (2005). Variability of familial risk of Alzheimer disease across the late life span.. Arch Gen Psychiatry.

[61] Mendez MF, McMurtray A (2006). Frontotemporal dementia-like phenotypes associated with presenilin-1 mutations.. Am J Alzheimers Dis Other Demen.

[62] Bernardi L, Tomaino C, Anfossi M, Gallo M, Geracitano S (2009). Novel PSEN1 and PGRN mutations in early-onset familial frontotemporal dementia.. Neurobiol Aging.

[63] Marcon G, Di Fede G, Giaccone G, Rossi G, Giovagnoli AR (2009). A novel Italian presenilin 2 gene mutation with prevalent behavioral phenotype.. J Alzheimers Dis.

[64] Wijsman EM, Pankratz ND, Choi Y, Rothstein JH, Faber KM (2011). Genome-wide association of familial late-onset Alzheimer's disease replicates BIN1 and CLU and nominates CUGBP2 in interaction with APOE.. PLoS genetics.

[65] McKhann G, Drachman D, Folstein M, Katzman R, Price D (1984). Clinical diagnosis of Alzheimer's disease: report of the NINCDS-ADRDA Work Group under the auspices of Department of Health and Human Services Task Force on Alzheimer's Disease.. Neurology.

[66] Brunak S, Engelbrecht J, Knudsen S (1991). Prediction of human mRNA donor and acceptor sites from the DNA sequence.. J Mol Biol.

