# Correction: Rare Variants in *APP*, *PSEN1* and *PSEN2* Increase Risk for AD in Late-Onset Alzheimer's Disease Families

**DOI:** 10.1371/annotation/c92e16da-7733-421d-b063-1db19488daa6

**Published:** 2012-05-10

**Authors:** Carlos Cruchaga, Sumitra Chakraverty, Kevin Mayo, Francesco L. M. Vallania, Robi D. Mitra, Kelley Faber, Jennifer Williamson, Tom Bird, Ramon Diaz-Arrastia, Tatiana M. Foroud, Bradley F. Boeve, Neill R. Graff-Radford, Pamela St. Jean, Michael Lawson, Margaret G. Ehm, Richard Mayeux, Alison M. Goate, for the NIA-LOAD/NCRAD Family Study Consortium

An author was inadvertently omitted from the by-line and Author Contributions section. Gabe Haller is second author of this manuscript. Gabe Haller's affiliation is: 1 Department of Psychiatry, Washington University, St. Louis, MO, USA. Author Contributions are: Analyzed the data and Contributed reagents/materials/analysis tools. 

