# Supplementary material for: Rare Variants in APP, PSEN1 and PSEN2 Increase Risk for AD in Late-Onset Alzheimer's Disease Families
Source: PLoS One. 2012 Feb 1;7(2):e31039. doi: 10.1371/journal.pone.0031039 (PMC3270040; doi:10.1371/journal.pone.0031039)
Supplement: Materials and Methods S1 — Additional material and methods. (DOC) [file pone.0031039.s001.doc]

**Rare variants in *APP*, *PSEN1* and *PSEN2* increase risk for AD in late-onset Alzheimer’s Disease families**

Carlos Cruchaga^1*^, Sumitra Chakraverty^1^, Kevin Mayo^1^, Francesco L.M. Vallania^2^, Robi D. Mitra^2^, Kelley Faber^3^, Jennifer Williamson^4^, Tom Bird^5^, Ramon Diaz-Arrastia^6^, Tatiana M. Foroud^3^, Bradley F. Boeve^7^, Neill R. Graff-Radford^8^, Pamela St. Jean^9^, Michael Lawson^9^, Margaret G. Ehm^9^, Richard Mayeux^4^, and Alison M. Goate^1,2*^ for the NIA-LOAD/NCRAD Family Study Consortium

1 Department of Psychiatry & Hope Center Program on Protein Aggregation and Neurodegeneration, Washington University, St. Louis, MO, USA

2 Department of Genetics, Washington University, St. Louis, MO, USA

3 Department of Medical and Molecular Genetics, Indiana University, Indianapolis

4 Taub Institute for Research on Alzheimer’s Disease and the Aging Brain, Columbia University College of Physicians and Surgeons, New York, New York

5 VA Medical Center and Departments of Neurology and Medicine, University of Washington, Seattle, WA, USA

6 Department of Neurology, University of Texas Southwestern Medical

Center, Dallas, Texas

7 Department of Neurology, Mayo Clinic, Rochester, Minnesota

8 Department of Neurology, Mayo Clinic, Jacksonville, Florida

9 Genetics, GlaxoSmithKline, Research Triangle Park, North Carolina

^*^To whom correspondence should be addressed at: Department of Psychiatry, Washington University School of Medicine, 660 South Euclid Avenue B8134, St. Louis, MO 63110. E-mail: [goatea@psychiatry.wustl.edu](mailto:goatea@psychiatry.wustl.edu), tel. 314-362-8691, fax. 314-747-2983 or [cruchagc@psychiatry.wustl.edu](mailto:cruchagc@psychiatry.wustl.edu) tel. 314-286-0546, fax. 314-362-2244

**METHODS**

**DNA sequencing and genotyping**

We used the next-generation, pooled-DNA method described by Druley et al., and Vallania et al. to identify sequence variants in *APP, PSEN1, PSEN2, MAPT* and *GRN*, and estimate variant frequency in the pooled DNA. An advantage of this approach is that it combines the pooled DNA approach with next-generation sequencing technology allowing accurate identification of sequence variants in several genes and hundreds of samples.

Genomic DNA was isolated using standard techniques and individually quantified by PicoGreen. Four different pools with 100 ng of DNA from 110 individuals were made. The coding exons and flanking regions were individually PCR amplified using specific primers and *Pfu* Ultra high-fidelity polymerase (Stratagene). An average of 20 diploid genomes (approximately 0.14 ng DNA) per individual were used as input into a total of 69 PCR reactions that covered 46,319 bases from the 5 genes. The PCR products were pooled in two different sets in equimolar ratios of PCR products, ligated and randomly fragmented. The first set of PCR products contained the amplicons for the *APP*, *PSEN1* and *GRN* genes. The second set of PCR products contained the amplicons for the *MAPT*, and *PSEN2* genes. The fragmented PCR products were prepared for sequencing in the Illumina Genome Analyzer II following the Illumina Genomic Library sample preparation protocol.

pCMV6 control DNA amplicon (1908 base pairs) was included in the reaction as a negative control. We used the first 900bp of the pCMV6 amplicon to model the errors across the 36-bp Illumina reads and to create an error model for each sequencing run. We also included, as positive controls ten different constructs with known mutations at a relative frequency of one mutated copy for 250 normal copies. This ratio was chosen because it is similar to the relative frequency expected for a single mutation in one pool (1 chromosome mutated in 110 samples= 1/220). The SPLINTER algorithm, including the specific error model for each run, was used to call and estimate the frequency of the sequence variants.

The fold coverage necessary to identify a singleton mutation in the pools was estimated by using the positive controls. We calculated that it was necessary to have 30 fold coverage or higher per nucleotide and sample to identify the singletons. The necessary number of lanes to obtain a minimum of 30 fold coverage per base and sample were run.

**Statistical Analysis**

Two-tailed t-tests and the Mann-Whitney U test were used to compare the age at onset (AAO) and the number of affected individuals between the different groups. The effect of variants on AAO was analyzed, using a Cox proportional hazards model (proc PHREG, SAS), including gender, and *APOE* genotype in the model and family as an aggregation term. Fisher’s exact test was used to compare the frequency of sequence variants identified in the sequenced LOAD samples to those found in the 14,196 non-selected individuals, and in the 1,000 genome project.

We used a logistic regression model to identify the variables that could predict the presence of a sequence variant in this series. All variants were included in the model independently of their pathogenicity. The variables that were included in the model were *APOE* genotype, gender, age at onset and the number of affected individuals in each family. In addition a stepwise option was included in order to identify the important variables. A ROC curve was also generated for the model including the significant variables.

**GSK study**

*Ethics Statement*

GlaxoSmithKline (GSK) collected human blood in collaborative research trials with investigators during 2002-2010 for other studies. All study participants in the component studies provided written informed consent for the use of their DNA in genetic studies and this was recorded via electronic case report.

# *Study sample*

A description of the subjects included in this study is provided in detail below. The primary selection criteria applied to each study was the availability of at least 10ug of DNA with a concentration at least 195 ng/ul from a primary blood sample.

## *Study descriptions*

**CoLaus Study**: A population-based study of 6,188 European white subjects age 35-75 years drawn from Lausanne Switzerland, through the CHUV University Hospital. Subjects included in the current study include 1,774 participants in the follow-on study of psychiatric traits (PsyCoLaus) and 772 extremes of several selected cardiovascular disease-associated traits. There was an overlap of 460 subjects between these two selections.

**LOLIPOP Study**: A population-based study of 21,915 subjects primarily of Indian Asians and Northwestern Europeans aged 35–75 years, identified from the lists of 58 general practitioners in West London. Subjects included in the current study include a random selection of 499 Indian Asians, 400 European whites selected for overlap with previous genome-wide genotyping studies, 149 European whites selected as extremes from several cardiovascular disease-related traits and 285 subjects of other non-European ancestry.

**Metabolic Syndrome GEMS Study**: The GEMS Study of Metabolic Syndrome and related traits included two types of samples; families and a set of unrelated cases and controls. Families (3384 individuals from 535 families) were recruited from six study sites located in Australia, Canada, Finland, Switzerland, Turkey and the United States. Eligible families consisted of a minimum of two siblings (an affected sib-pair) with atherogenic dyslipidemia (ADL). In the case-control arm, a set of approximately 1,000 cases with ADL and 1,000 normolipidemic controls were recruited from the same GEMS sites. Details of the recruitment procedures, subject characteristics, and inclusion/exclusion criteria for both the family and case control studies have been previously described. The current study includes 1,570 unrelated cases and controls and 30 parent-offspring trios for assessing sequence data quality.

**Coronary Artery Disease (CAD) MedStar Study**: A premature CAD collection designed to investigate the genetics of plaque stability in acute coronary syndrome (ACS). The full study is comprised of 452 ACS CAD cases, 491 non-ACS CAD cases, and 483 non-CAD controls. Subjects were identified prospectively from the patient population of the Cardiovascular Research Institute (MedStar/Washington Hospital Center). Standard criteria were used to identify cases with myocardial infarction and cases diagnosed with clinically significant coronary atherosclerosis without myocardial infarction. Subjects included in the current study include a selection of 609 ACS and non-ACS CAD cases.

**Irritable Bowel Syndrome Study**: A population-based study of 678 cases and 539 controls from 3 recruitment sites in Canada and the United States. Cases were deeply phenotyped with a history of irritable bowel syndrome (IBS) for at least 6 months confirmed by a physician and according to the Rome II criteria and either a colonoscopy/barium enema with normal results supporting IBS diagnosis. Controls were matched to IBS cases and had no previous IBS diagnosis. The current study includes 317 cases.

**Genetics of Rheumatoid Arthritis (GORA) Study**: Patients with rheumatoid arthritis were recruited from six centers (Manchester, Sheffield, Leeds, Aberdeen, Oxford and London) across the United Kingdom as described previously. Cases (~1,000) were of Northern European descent and all fulfilled the 1987 American College of Rheumatology classification criteria. A similar number of healthy controls were recruited from five of the six centers. The current study includes 615 cases.

**Multiple Sclerosis genMSA Study**: A study of 1005 thoroughly phenotyped multiple sclerosis (MS) cases and 1,012 matched controls primarily of European ancestry from three sites in the United States, the Netherlands and Switzerland. The current study includes 673 cases.

**Multiple Sclerosis African American Study**: A study of African American cases and controls with subjects recruited from 39 states. Cases were characterized through a systematic medical record review. Controls were invited to participate in the study by the probands and constitute primarily non-consanguinous spouses or friends of MS patients. All study participants were self-reported African-Americans and ancestry was documented based on genotyping results of 186 informative SNPs. The current study included 340 cases and 260 controls.

**Epilepsy HitDIP Study**: A study of 719 cases and 687 controls recruited from Norway and Finland. All patients had a definite diagnosis of epilepsy according to International League Against Epilepsy (ILAE) definitions. Controls had no neuropsychiatric condition. The current study includes 185 Finnish cases.

**Epilepsy GenEpa Study**: A study of 318 cases and 348 controls from Swiss Epilepsy Centre, Zurich. All patients had a definite diagnosis of epilepsy according to ILAE definitions. Controls had no neuropsychiatric condition. The current study includes 125 cases.

**Unipolar Depression Study**: A study of 1,000 cases recruited from three ascertainment sites in Southern Germany (Munich, Augsburg and Ingolstadt) and 1,029 controls ascertained by the Max Plank Institute of Psychiatry in Munich. Cases were diagnosed with recurrent major depressive disorder and controls were age and gender-matched non-affected controls . The current study includes 775 cases.

**Schizophrenia Study**: A study of approximately 1,600 cases and 850 controls collected from four sites in Aberdeen, UK, Greenock, UK, Munich, Germany and Quebec City, Canada. Cases were diagnosed with schizophrenia according to DSM-IV or ICD-10 criteria and healthy volunteers were randomly selected from the general population. The current study includes 1,109 cases.

**Bipolar Disorder Study**: A study of 965 bipolar cases and 933 controls of European ancestry from a multicenter study from the Centre for Addiction and Mental Health in Toronto, Canada, the Institute of Psychiatry in London, UK and the University of Dundee, UK. Each case was assessed when euthymic and had to have been diagnosed (lifetime) with the DSM-IV/ICD-10 bipolar I or bipolar II disorder. The current study includes 786 cases.

**Chronic Obstructive Pulmonary Disease ECLIPSE Study**: ECLIPSE (Evaluation of COPD Longitudinally to Identify Predictive Surrogate End-points) is a three-year non-interventional longitudinal prospective study being conducted at 46 centers in 12 countries and is comprised of clinically relevant COPD individuals with Global Initiative for Chronic Obstructive Lung Disease (GOLD) stage 11_IV COPD with a number of smoking and non-smoking and non-disease controls. The current study includes 1,002 cases from ten countries.

**COPD HitDIP Study**: A study of approximately 1,000 cases and 1,000 controls from Bergen, Norway. Cases consist of α1-antitrypsin deficiency-negative individuals with moderate to severe COPD according to GOLD criteria. The current study includes 782 cases.

Of the above subjects, we sent the data for 12,481 subjects, passing QC, to UCLA for generation of an allele frequency table. Of these subjects, there were 594 African Americans, 566 South Asians, 29 Hispanic, 84 Ashkenazi Jews, 241 of mixed ethnicity and 10967 white Caucasians.

*Age and Sex*

See table S5 for demographic statistics for the different studies

# *Sequencing methods Genotype calling*

The high-depth sequence data were generated by BGI (Shenzhen, China) by sequencing the exons plus 50 bp of flanking sequence for 202 genes resulting in approximately 850 kb of coding and 323 kb of noncoding (untranslated) exons. Candidate variants were identified for each sample where a genotype was called with a minimum sequencing depth of four, a minimum consensus quality of 20 with no other variants within four base pairs. Genotypes were called in all samples for all variant positions identified by aggregating all sequenced samples. Consensus genotypes were called at these position bases for each sample with a minimum depth of seven and minimum consensus quality of 20. The median sequence depth per sample was 27x. Samples were excluded from the data if 1) their average sequencing depth was less than 10, 2) sequence-based genotypes were more than 15% discordant with genome-wide panel genotypes or 3) the sample was sequenced multiple times and had lower average sequencing depth. Analysis of 133 sample duplicates resulted in a discordance rate among heterozygous genotype calls of 0.90% with lower rates in more common variants. An overall heterozygote genotype error rate was estimated to be 0.50%. These quality settings resulted in median genotype missingness of 0.0069, 0.011, 0.014, and 0.021 for variants with MAF<0.001, 0.001<MAF<0.005, 0.005<MAF<0.05, MAF>0.05. SNVs were considered deleterious if they were predicted to be damaging by SIFT, PolyPhen or at a position with a phyloP conservation score ≥ 2.

**1,000 genome project**

We used the 1,000 genome project (release Oct. 2010) to compare the frequency of rare (MAF<0.05) non-synonymous and splice-site sequence variants in our study with unselected populations from the 1,000 genome project. In the 1,000 genome project samples for at least 3 different populations were included, therefore it is expected that there would be more rare variants due to ethnicity. We included these populations in the analyses because the AD study population also includes samples of different ethnicities. *APP, PSEN2* and *GRN* were sequenced only in pilots 1 and 2 of the 1,000 genome project. *PSEN1* and *MAPT* were also sequenced in pilot 3. Pilot 1 includes sequencing data from 60 Utah residents with Northern and Western European ancestry from the CEPH collection (CEU), 60 Japanese in Tokyo, Japan and Han Chinese in Beijing, China (CHBJPT) and 60 Yorubans in Ibadan, Nigeria (YRI). Pilot 2 includes sequencing data from a CEU trio and a YRI trio. Finally pilot 3 includes sequencing data from 90 CEU, 66 Tuscans in Italy (TSI), 109 CHB, 107 Chinese in Metropolitan Denver, Colorado (CHD), 105 JPT, 108 Luhya in Webuye, Kenya (LWK) and 112 YRI.

**REFERENCES**

1. Druley TE, Vallania FL, Wegner DJ, Varley KE, Knowles OL, et al. (2009) Quantification of rare allelic variants from pooled genomic DNA. Nat Methods 6: 263-265.

2. Vallania FL, Druley TE, Ramos E, Wang J, Borecki I, et al. (2010) High-throughput discovery of rare insertions and deletions in large cohorts. Genome Res 20: 1711-1718.

3. Zhao H (2000) Family-based association studies. Stat Methods Med Res 9: 563-587.

4. Blacker D, Wilcox MA, Laird NM, Rodes L, Horvath SM, et al. (1998) Alpha-2 macroglobulin is genetically associated with Alzheimer disease. Nat Genet 19: 357-360.

5. Siegmund KD, Langholz B, Kraft P, Thomas DC (2000) Testing linkage disequilibrium in sibships. Am J Hum Genet 67: 244-248.

6. Kraft P, Thomas DC (2004) Case-sibling gene-association studies for diseases with variable age at onset. Stat Med 23: 3697-3712.

7. Romas SN, Mayeux R, Tang MX, Lantigua R, Medrano M, et al. (2000) No association between a presenilin 1 polymorphism and Alzheimer disease. Archives of Neurology 57: 699-702.

8. Cruchaga C, Kauwe JS, Mayo K, Spiegel N, Bertelsen S, et al. (2010) SNPs Associated with Cerebrospinal Fluid Phospho-Tau Levels Influence Rate of Decline in Alzheimer's Disease. PLoS Genet 6.

9. Cruchaga C, Graff C, Chiang HH, Wang J, Hinrichs AL, et al. (2011) Association of TMEM106B Gene Polymorphism With Age at Onset in Granulin Mutation Carriers and Plasma Granulin Protein Levels. Arch Neurol.

10. Firmann M, Mayor V, Vidal PM, Bochud M, Pecoud A, et al. (2008) The CoLaus study: a population-based study to investigate the epidemiology and genetic determinants of cardiovascular risk factors and metabolic syndrome. BMC cardiovascular disorders 8: 6.

11. Preisig M, Waeber G, Vollenweider P, Bovet P, Rothen S, et al. (2009) The PsyCoLaus study: methodology and characteristics of the sample of a population-based survey on psychiatric disorders and their association with genetic and cardiovascular risk factors. BMC psychiatry 9: 9.

12. Kooner JS, Chambers JC, Aguilar-Salinas CA, Hinds DA, Hyde CL, et al. (2008) Genome-wide scan identifies variation in MLXIPL associated with plasma triglycerides. Nature Genetics 40: 149-151.

13. Ling H, Waterworth DM, Stirnadel HA, Pollin TI, Barter PJ, et al. (2009) Genome-wide linkage and association analyses to identify genes influencing adiponectin levels: the GEMS Study. Obesity 17: 737-744.

14. Wyszynski DF, Waterworth DM, Barter PJ, Cohen J, Kesaniemi YA, et al. (2005) Relation between atherogenic dyslipidemia and the Adult Treatment Program-III definition of metabolic syndrome (Genetic Epidemiology of Metabolic Syndrome Project). The American journal of cardiology 95: 194-198.

15. Assimes TL, Holm H, Kathiresan S, Reilly MP, Thorleifsson G, et al. (2010) Lack of association between the Trp719Arg polymorphism in kinesin-like protein-6 and coronary artery disease in 19 case-control studies. Journal of the American College of Cardiology 56: 1552-1563.

16. Vignal C, Bansal AT, Balding DJ, Binks MH, Dickson MC, et al. (2009) Genetic association of the major histocompatibility complex with rheumatoid arthritis implicates two non-DRB1 loci. Arthritis and rheumatism 60: 53-62.

17. Baranzini SE, Wang J, Gibson RA, Galwey N, Naegelin Y, et al. (2009) Genome-wide association analysis of susceptibility and clinical phenotype in multiple sclerosis. Human molecular genetics 18: 767-778.

18. Oksenberg JR, Barcellos LF, Cree BA, Baranzini SE, Bugawan TL, et al. (2004) Mapping multiple sclerosis susceptibility to the HLA-DR locus in African Americans. American journal of human genetics 74: 160-167.

19. Cree BA, Reich DE, Khan O, De Jager PL, Nakashima I, et al. (2009) Modification of Multiple Sclerosis Phenotypes by African Ancestry at HLA. Archives of Neurology 66: 226-233.

20. Patterson N, Hattangadi N, Lane B, Lohmueller KE, Hafler DA, et al. (2004) Methods for high-density admixture mapping of disease genes. American journal of human genetics 74: 979-1000.

21. Heinzen EL, Radtke RA, Urban TJ, Cavalleri GL, Depondt C, et al. (2010) Rare deletions at 16p13.11 predispose to a diverse spectrum of sporadic epilepsy syndromes. American journal of human genetics 86: 707-718.

22. Kasperaviciute D, Catarino CB, Heinzen EL, Depondt C, Cavalleri GL, et al. (2010) Common genetic variation and susceptibility to partial epilepsies: a genome-wide association study. Brain : a journal of neurology 133: 2136-2147.

23. Muglia P, Tozzi F, Galwey NW, Francks C, Upmanyu R, et al. (2010) Genome-wide association study of recurrent major depressive disorder in two European case-control cohorts. Molecular Psychiatry 15: 589-601.

24. Francks C, Tozzi F, Farmer A, Vincent JB, Rujescu D, et al. (2010) Population-based linkage analysis of schizophrenia and bipolar case-control cohorts identifies a potential susceptibility locus on 19q13. Molecular Psychiatry 15: 319-325.

25. Vestbo J, Anderson W, Coxson HO, Crim C, Dawber F, et al. (2008) Evaluation of COPD Longitudinally to Identify Predictive Surrogate End-points (ECLIPSE). The European respiratory journal : official journal of the European Society for Clinical Respiratory Physiology 31: 869-873.

26. Pillai SG, Ge D, Zhu G, Kong X, Shianna KV, et al. (2009) A genome-wide association study in chronic obstructive pulmonary disease (COPD): identification of two major susceptibility loci. PLoS genetics 5: e1000421.

27. Ng PC, Henikoff S (2003) SIFT: Predicting amino acid changes that affect protein function. Nucleic Acids Res 31: 3812-3814.

28. Ramensky V, Bork P, Sunyaev S (2002) Human non-synonymous SNPs: server and survey. Nucleic Acids Res 30: 3894-3900.
