# Supplementary material for: Rare Variants in APP, PSEN1 and PSEN2 Increase Risk for AD in Late-Onset Alzheimer's Disease Families
Source: PLoS One. 2012 Feb 1;7(2):e31039. doi: 10.1371/journal.pone.0031039 (PMC3270040; doi:10.1371/journal.pone.0031039)
Supplement: Results S1 — Additional results. (DOC) [file pone.0031039.s002.doc]

**Rare variants in *APP*, *PSEN1* and *PSEN2* increase risk for AD in late-onset Alzheimer’s Disease families**

Carlos Cruchaga^1*^, Sumitra Chakraverty^1^, Kevin Mayo^1^, Francesco L.M. Vallania^2^, Robi D. Mitra^2^, Kelley Faber^3^, Jennifer Williamson^4^, Tom Bird^5^, Ramon Diaz-Arrastia^6^, Tatiana M. Foroud^3^, Bradley F. Boeve^7^, Neill R. Graff-Radford^8^, Pamela St. Jean^9^, Michael Lawson^9^, Margaret G. Ehm^9^, Richard Mayeux^4^, and Alison M. Goate^1,2*^ for the NIA-LOAD/NCRAD Family Study Consortium

1 Department of Psychiatry & Hope Center Program on Protein Aggregation and Neurodegeneration, Washington University, St. Louis, MO, USA

2 Department of Genetics, Washington University, St. Louis, MO, USA

3 Department of Medical and Molecular Genetics, Indiana University, Indianapolis

4 Taub Institute for Research on Alzheimer’s Disease and the Aging Brain, Columbia University College of Physicians and Surgeons, New York, New York

5 VA Medical Center and Departments of Neurology and Medicine, University of Washington, Seattle, WA, USA

6 Department of Neurology, University of Texas Southwestern Medical

Center, Dallas, Texas

7 Department of Neurology, Mayo Clinic, Rochester, Minnesota

8 Department of Neurology, Mayo Clinic, Jacksonville, Florida

9 Genetics, GlaxoSmithKline, Research Triangle Park, North Carolina

^*^To whom correspondence should be addressed at: Department of Psychiatry, Washington University School of Medicine, 660 South Euclid Avenue B8134, St. Louis, MO 63110. E-mail: [goatea@psychiatry.wustl.edu](mailto:goatea@psychiatry.wustl.edu), tel. 314-362-8691, fax. 314-747-2983 or [cruchagc@psychiatry.wustl.edu](mailto:cruchagc@psychiatry.wustl.edu) tel. 314-286-0546, fax. 314-362-2244

**RESULTS**

**DNA sequencing and genotyping**

We used the positive and the negative controls to estimate the sensitivity and specificity of the next-generation pooled sequencing reaction. All of the variants present in the positive control were successfully identified, giving a sensitivity of 100%. An additional 3 variants in the positive control and four in the negative control were also called by the SPLINTER software, which indicates that the specificity is 99.9 (Table S1). These data indicate that by using the next-generation pooled DNA method we should have been able to identify all of the singleton variants present in this series.

**Statistical Analysis. Logistic regression.**

We found no difference in the frequency of *APOE ε4*, or in the gender proportions between the carriers for a sequence variant and the non-carriers (Table S3, figures S1-S3). Neither *APOE* genotype nor gender were good predictors of the presence of a sequence variant. In the stepwise selection for the logistic regression the number of affected individuals and the age at onset were predictors of variant carrier status. The most important variable was the number of affected individuals in a family (p=0.0026, p-value corrected for the onset); OR 1.15; 95%CI=1.052-1.267). The age at onset was also significant after correcting for the number of affected individuals in a family (p=0.02; OR 0.96; 95%CI=0.926-0.996).

Although the number of affected individuals and the age at onset of the sequenced individual are significantly associated with the presence of a sequence variant these variables or the combination of both are not good predictors of the presence of a sequence variant. The model containing the number of affected individuals and the onset only explains 7.47% of the variability in the model. The area under the ROC curve (c-statistic for the mode) (figure S3) was 0.634, confirming that these variables are not very good predictors for the presence of a sequence variant.

Similar results were obtained when only the pathogenic mutations were studied (data not shown).

**Non-pathogenic or likely non-pathogenic variants.**

We also identified a family heterozygous for the MAPT-R5H variant, which has been classified as pathogenic by the AD&FTD mutation database. This mutation was found in a Japanese family with eight affected individuals, but only a single affected individual was a mutation carrier suggesting that this variant in *MAPT* is not pathogenic. The *MAPT* A239T (exon 9) and V224G (exon 4A) variants were found in 4 and 2 families respectively. Both variants were present in all affected individuals suggesting that these variants may be pathogenic. However both variants were also found in unrelated controls. Two additional variants in *MAPT* (R168C (exon 4A) and A152T (exon 7)) and two in *GRN* (P85A and T268M) are unlikely to be pathogenic based on segregation data (Table S3). The *APP* E599K variant was found in two sequenced samples, one of which had a definite diagnosis of LOAD. Among the other family members, this variant was found in three of four additional affected individuals. This variant was also found in 42 additional samples (2 cases and 2 controls from the Washington University series and 38 individuals in the GSK study), suggesting that it is not pathogenic (Table 5).
