# Supplementary material for: Rare Variants in APP, PSEN1 and PSEN2 Increase Risk for AD in Late-Onset Alzheimer's Disease Families
Source: PLoS One. 2012 Feb 1;7(2):e31039. doi: 10.1371/journal.pone.0031039 (PMC3270040; doi:10.1371/journal.pone.0031039)
Supplement: Table S1 — Sensitivity and specificity of the next-gen DNA approach. (DOC) [file pone.0031039.s003.doc]

| **Table S1: Sensitivity and specificity of the next-gen DNA approach** | | | | |
| --- | --- | --- | --- | --- |
|  | **SNPs called** | **True SNPs** | **False SNPs** | **True negatives** |
| **Positive controls** | 78 | 75 | 3 | 1262 |
| **Negative controls** | 4 | 0 | 4 | 7628 |
| •Using the first 20 bases | |  |  |  |
| –Sensitivity = 100% | |  |  |  |
| –Specificity = 99.9% | |  |  |  |
| The negative and the positive controls were used to calculate the sensitivity and specificity of the method. | | | | |
