# Supplementary material for: Rare Variants in APP, PSEN1 and PSEN2 Increase Risk for AD in Late-Onset Alzheimer's Disease Families
Source: PLoS One. 2012 Feb 1;7(2):e31039. doi: 10.1371/journal.pone.0031039 (PMC3270040; doi:10.1371/journal.pone.0031039)
Supplement: Table S2 — List of all non-pathogenic and unknown pathogenicity rare non-synonymous, splice site and nonsense sequence variants identified. (DOC) [file pone.0031039.s004.doc]

| **Table S2:** List of all non-pathogenic and unknown pathogenicity rare non-synonymous, splice site and nonsense sequence variants identified. | | | | | | | | | | | | | |
| --- | --- | --- | --- | --- | --- | --- | --- | --- | --- | --- | --- | --- | --- |
| **Gene** | **Change** | **# of fam.** | **Status** | **Polyphen2** | | | | **Affected** | | **Unaffected** | | | |
|  |  |  |  |  |  |  |  | **Carriers** | **Non-carriers** | **Carriers** | | **Non-carriers** | |
| **Non-pathogenic or likely non-pathogenic** | | | | | | | | |  |  | |  | |
| ***APP*** | G322A | 1 | Novel | | probably damaging | | 4 | | 7 | 1 | | 2 | |
|  | E599K | 2 | Novel | | probably damaging | | 4 | | 1 | 2 | | 6 | |
|  | A673T | 1 | Previously reported Not pathogenic | | benign | | 1 | | 2 | 0 | | 1 | |
| ***PSEN2*** | R62H | 6 | Previously reported Unclear | | benign | | 12 | | 8 | 8 | | 35 | |
|  | R71W | 6 | Previously reported Pathogenic | | probably damaging | | 6 | | 8 | 3 | | 10 | |
|  | M174V | 1 | Previously reported Pathogenic | | benign | | 1 | | 4 | 3 | | 17 | |
| ***MAPT*** | R5H | 1 | Previously reported Pathogenic | | probably damaging | | 1 | | 1 | 4 | | 1 | |
|  | R168C | 1 | Novel | | benign | | 1 | | 4 | 3 | | 28 | |
|  | A152T | 5 | Novel | | probably damaging | | 8 | | 5 | 2 | | 16 | |
|  | V224G | 2 | Novel | | possibly damaging | | 6 | | 0 | 1 | | 2 | |
|  | A239T | 4 | Novel | | benign | | 6 | | 0 | 3 | | 4 | |
| ***GRN*** | P85A | 2 | Novel | | | probably damaging | 3 | | 3 | 0 | | 1 | |
|  | V141I | 1 | Previously reported Not pathogenic | | | benign | 2 | | 1 | 1 | | 2 | |
|  | T268M | 1 | Novel | | | benign | 1 | | 2 | 5 | | 7 | |
|  | A324T | 1 | Previously reported Not pathogenic | | | benign | 2 | | 1 | 2 | | 0 | |
|  | D376N | 1 | Previously reported No pathogenic | | | benign | 1 | | 0 | 0 | | 0 | |
|  | R433Q | 1 | Previously reported Not pathogenic | | | benign | 1 | | 0 | 0 | | 0 | |
| **Unknown** | | | | | | | | | | | | | |
| ***APP*** | G191E | 1 | Novel | | | probably damaging | | 1 | 0 | | 0 | | 0 |
|  | V340M | 1 | Novel | | | probably damaging | | 2 | 1 | | 1 | | 1 |
| ***PSEN1*** | P7L | 1 | Novel | | | benign | | 2 | 1 | | 0 | | 5 |
| ***MAPT*** | S427F | 1 | Novel | | | probably damaging | | 2 | 1 | | 1 | | 0 |
| ***GRN*** | D135V | 1 | Novel | | | possibly damaging | | 1 | 0 | | 0 | | 0 |
|  | M207T | 1 | Novel | | | benign | | 1 | 1 | | 0 | | 1 |
|  | V514M | 1 | Novel | | | possibly damaging | | 1 | 0 | | 0 | | 0 |
|  | V519M | 2 | Novel | | | probably damaging | | 2 | 0 | | 1 | | 0 |
| List of the non-synonymous, splice-site and nonsense variants identified in the 439 sequenced samples. The identified variants were genotyped in all available family samples. The number of affected carriers, non-carriers and the un-affected carriers, non-carriers, as well as the mean age at onset and the standard deviation for the affected and the age at the last assessment for the unaffected individuals are shown.  The variants were classified as pathogenic, non-pathogenic or unknown based on our segregation analyses and previous reports | | | | | | | | | | | | | |
