# Supplementary material for: Rare Variants in APP, PSEN1 and PSEN2 Increase Risk for AD in Late-Onset Alzheimer's Disease Families
Source: PLoS One. 2012 Feb 1;7(2):e31039. doi: 10.1371/journal.pone.0031039 (PMC3270040; doi:10.1371/journal.pone.0031039)
Supplement: Table S3 — Association of APOE with AAO. (DOC) [file pone.0031039.s005.doc]

| **Table S3: Association of APOE with AAO** | |
| --- | --- |
|  | **p-value** |
| All affected samples | 1.42x10^-15^ |
| Sequenced samples | 2.02x10^-6^ |
| Families with sequence variants | 6.13x10^-3^ |
| Families without sequence variants | 1.06x10^-14^ |
| Age at onset were analyzed for association with the number of *APOE 4* alleles by the Kaplan-Meier method and tested for significant differences, using a proportional hazards model (proc PHREG, SAS). Family and gender were included in the model to take into account the relatedness between samples and the potential differences in age at onset between mutations. | |
