# Supplementary material for: Rare Variants in APP, PSEN1 and PSEN2 Increase Risk for AD in Late-Onset Alzheimer's Disease Families
Source: PLoS One. 2012 Feb 1;7(2):e31039. doi: 10.1371/journal.pone.0031039 (PMC3270040; doi:10.1371/journal.pone.0031039)
Supplement: Table S4 — Comparison of the sequenced samples and families with and without variants. (DOC) [file pone.0031039.s006.doc]

| **Table S4: Comparison of the sequenced samples and families with and without variants.** | | | | | | | | | | | | |
| --- | --- | --- | --- | --- | --- | --- | --- | --- | --- | --- | --- | --- |
|  | **Sequenced samples** | | | | |  | **All family samples** | | | | |  |
|  | **Variant Carriers** | |  | **Non-Carriers** | |  | **Variant Carriers** | |  | **Non-Carriers** | |  |
|  | **n** | **%** |  | **n** | **%** | **p** | **n** | **%** |  | **n** | **%** | **P** |
| **APOE4+** | 52 | 71.15 |  | 332 | 72.29 | 0.86 | 164 | 61.51 |  | 864 | 71.76 | **0.0091** |
|  |  |  |  |  |  |  |  |  |  |  |  |  |
|  | **n** | **Mean ± SD**  **(Range)** |  |  | **Mean ± SD**  **(Range)** | **p** | **n** | **Mean ± SD (Range)** |  | **n** | **Mean ± SD (Range)** | **p** |
| **AAO** | 60 | 67.80 ± 8.96  (42-85) |  | 376 | 70.51 ± 8.05  (30-92) | **0.004** | 266 | 71.81 ± 6.17  (60-85) |  | 1507 | 72.97 ± 5.51  (60-85) | **0**.19 |
|  |  |  |  |  |  |  |  |  |  |  |  |  |
| **# of**  **Affected** | - | - |  | - | - |  | 60 | 6.88 ± 4.5  (4-28) |  | 376 | 5.62 ± 2.21  (4-19) | **0.0008** |
| The *APOE4* frequency was compared in sequenced samples with and without variants and in the families with variants vs the families without variants.  No significant difference in the % of individuals with *APOE4* was found between the carriers for any identified sequence variant compared with the non-carriers among the sequenced samples.  The frequency of APOE4+ individuals for all the individuals in families in which the sequenced sample carried a sequence variant is significantly lower than all the individuals from the families for which the sequenced sample did not carry a sequence variant.  Among the sequenced samples the carriers of sequence variants have an earlier AAO than the non-carriers; however the mean AAO for the carrier families is not significantly different than the non-carriers.  The number of affected individuals is significantly higher in the variant carrying families compared with the non-carriers. | | | | | | | | | | | | |
