# Supplementary material for: Rare Variants in APP, PSEN1 and PSEN2 Increase Risk for AD in Late-Onset Alzheimer's Disease Families
Source: PLoS One. 2012 Feb 1;7(2):e31039. doi: 10.1371/journal.pone.0031039 (PMC3270040; doi:10.1371/journal.pone.0031039)
Supplement: Table S5 — Summary of sample characteristics for the GSK study. (DOC) [file pone.0031039.s007.doc]

| **Table S5: Summary of sample characteristics for the GSK study** | | | | | |
| --- | --- | --- | --- | --- | --- |
| **Study** | **Number** | **Age (years)** | | | **Sex (% Female**) |
|  |  | **Mean** | **Median** | **Range** |  |
| **Population controls** | 3,384 | 50.6 | 49.8 | 23-80 | 47.20% |
| **Metabolic Syndrome** | 1,643 | 52.3 | 52.4 | 20-83 | 40.80% |
| **CAD** | 6,04 | 54.5 | 55 | 25-84 | 29.50% |
| **IBS** | 3,17 | 41.8 | 43.2 | 18-78 | 81.70% |
| **RA** | 611 | 62.2 | 62.8 | 20-90 | 72.80% |
| **MS** | 1,261 | 46.3 | 46 | 18-86 | 68.80% |
| **Epilepsy** | 275 | 44 | 43.5 | 18-76 | 55.60% |
| **Unipolar** | 742 | 51.5 | 51.1 | 19-88 | 67.50% |
| **Schizophrenia** | 1,099 | 42.2 | 41.5 | 18-81 | 27.90% |
| **Bipolar** | 777 | 46.6 | 46.9 | 18-83 | 65.00% |
| **COPD** | 1,768 | 63.3 | 64 | 40-91 | 38.90% |
| **Total** | **12,481** | **51.7** | **52.1** | **18-91** | **49.50%** |
| Metabolic Syndrome: 80 trios, 782 cases, 781 controls  MS: Includes 670 European cases, 339 African American cases, 252 African American controls.  Population controls: 2059 CoLaus, 1322 LOLIPOP, 3 Nigerian samples from 1000 Genome. | | | | | |
