# Supplementary material for: Rare Variants in APP, PSEN1 and PSEN2 Increase Risk for AD in Late-Onset Alzheimer's Disease Families
Source: PLoS One. 2012 Feb 1;7(2):e31039. doi: 10.1371/journal.pone.0031039 (PMC3270040; doi:10.1371/journal.pone.0031039)
Supplement: Figure S1 — The number of APOE 4 alleles is associated with age at onset. A) Age at onset was analyzed for association with the number of APOE 4 alleles in all the affected family members using the Kaplan-Meier method and tested for significant differences, using a proportional hazards model (proc PHREG, SAS). Family and gender were included in the model to take into account the relatedness between samples. Carriers of APOE 4 alleles have an earlier AAO than non-carriers B) Age at onset was analyzed for association with the number of APOE 4 alleles in the sequenced samples using the Kaplan-Meier method and tested for significant differences, using a proportional hazards model (proc PHREG, SAS). Carriers of APOE 4 alleles have an earlier AAO than non-carriers. (DOC) [file pone.0031039.s008.doc]

**Figure S1**

**
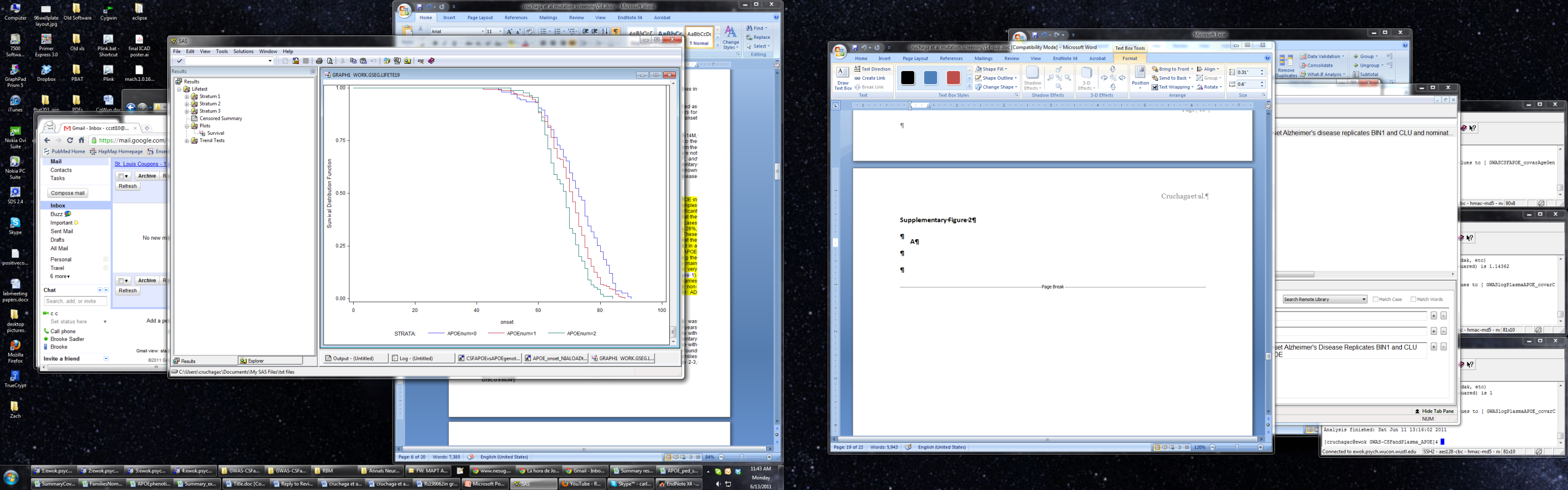

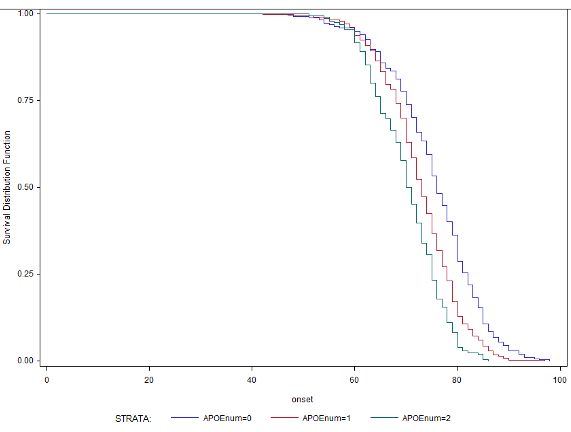
**

**A**

**B**

**p=3.53x10^-17^ n=1094**

**p=5.05x10^-5^ n=386**

**The number of *APOE 4* alleles is associated with age at onset A)** Age at onset was analyzed for association with the number of *APOE 4* alleles in all the affected family members using the Kaplan-Meier method and tested for significant differences, using a proportional hazards model (proc PHREG, SAS). Family and gender were included in the model to take into account the relatedness between samples. Carriers of *APOE 4* alleles have an earlier AAO than non-carriers **B)** Age at onset was analyzed for association with the number of *APOE 4* alleles in the sequenced samples using the Kaplan-Meier method and tested for significant differences, using a proportional hazards model (proc PHREG, SAS). Carriers of *APOE 4* alleles have an earlier AAO than non-carriers.
