# Supplementary material for: Rare Variants in APP, PSEN1 and PSEN2 Increase Risk for AD in Late-Onset Alzheimer's Disease Families
Source: PLoS One. 2012 Feb 1;7(2):e31039. doi: 10.1371/journal.pone.0031039 (PMC3270040; doi:10.1371/journal.pone.0031039)
Supplement: Figure S2 — Number of affected individuals in the families with any sequence variant compared to the families with no sequence variants. The families for which the selected sample carried a sequence variant have a higher mean number of affected individuals (6.88±4.5 (4–28)), than the families without sequence variants (5.62±2.21 (4–19)). For this analysis we included all of the sequence variants identified in this study, even if they were considered non-pathogenic. (DOC) [file pone.0031039.s009.doc]

**Figure S2**

**Number of affected individuals in the families with any sequence variant compared to the families with no sequence variants**. The families for which the selected sample carried a sequence variant have a higher mean number of affected individuals (6.88 ± 4.5 (4-28)), than the families without sequence variants (5.62 ± 2.21 (4-19)). For this analysis we included all of the sequence variants identified in this study, even if they were considered non-pathogenic.
