# Supplementary material for: Rare Variants in APP, PSEN1 and PSEN2 Increase Risk for AD in Late-Onset Alzheimer's Disease Families
Source: PLoS One. 2012 Feb 1;7(2):e31039. doi: 10.1371/journal.pone.0031039 (PMC3270040; doi:10.1371/journal.pone.0031039)
Supplement: Figure S3 — The number of affected individuals and the age at onset of the sequenced individual, but not gender or APOE genotype are associated with the presence of non-synonymous, nonsense or splice-site variants in the APP, PSEN1, PSEN2, MAPT or GRN genes. We used a logistic regression model to analyze the association of the number of affected individuals, the age at onset of the sequenced individual, gender and APOE genotype with the presence of sequence variants. The Odds Ratio (OR) and the 95% confidence interval were calculated. The risk of having a sequence variant in these genes increase by 1.33 (95%: 1.105–1.69) for every two affected individuals in the family. The risk of having a sequence variant in these genes decreased by 0.807 (95%: 0.67–0.96) for every five years increase in age at onset. (DOC) [file pone.0031039.s010.doc]

**Figure S3**


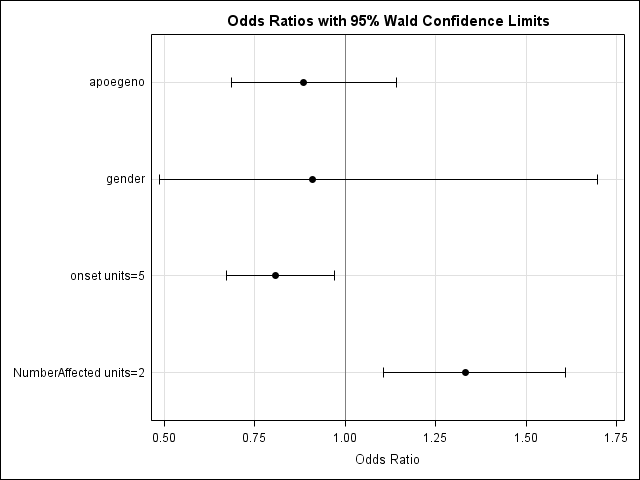


**The number of affected individuals and the age at onset of the sequenced individual, but not gender or *APOE* genotype are associated with the presence of non-synonymous, nonsense or splice-site variants in the *APP, PSEN1, PSEN2, MAPT* or *GRN* genes.** We used a logistic regression model to analyze the association of the number of affected individuals, the age at onset of the sequenced individual, gender and *APOE* genotype with the presence of sequence variants. The Odds Ratio (OR) and the 95% confidence interval were calculated. The risk of having a sequence variant in these genes increase by 1.33 (95%: 1.105-1.69) for every two affected individuals in the family. The risk of having a sequence variant in these genes decreased by 0.807 (95%: 0.67-0.96) for every five years increase in age at onset.
