# Supplementary material for: Rare Variants in APP, PSEN1 and PSEN2 Increase Risk for AD in Late-Onset Alzheimer's Disease Families
Source: PLoS One. 2012 Feb 1;7(2):e31039. doi: 10.1371/journal.pone.0031039 (PMC3270040; doi:10.1371/journal.pone.0031039)
Supplement: Figure S4 — ROC curve for the logistic regression model including the number of affected individuals in a family and age at onset for the presence or absence of sequence variations in the APP, PSEN1, PSEN2, MAPT or GRN genes. We used a logistic regression model to generate a ROC curve including the variables that could predict the presence of a sequence variant in this series. A stepwise regression analyses was used to include the most significant variables among: APOE genotype, gender, age at onset and the number of affected individuals in each family. The logistic regression identified age at onset (p = 0.0008; Area under the Curve = 0.6022) in the first step and number of affected individuals (p = 0.0001; Area under the Curve = 0.5779) in the second step. No other variable entered in the model. (DOC) [file pone.0031039.s011.doc]

**Figure S4**


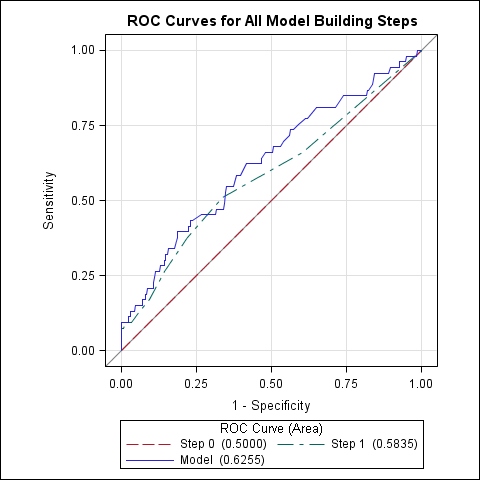


**ROC curve for the logistic regression model including the number of affected individuals in a family and age at onset for the presence or absence of sequence variations in the *APP*, *PSEN1*, *PSEN2*, *MAPT* or *GRN* genes.** We used a logistic regression model to generate a ROC curve including the variables that could predict the presence of a sequence variant in this series. A stepwise regression analyses was used to include the most significant variables among: *APOE* genotype, gender, age at onset and the number of affected individuals in each family. The logistic regression identified age at onset (p=0.0008; Area under the Curve=0.6022) in the first step and number of affected individuals (p=0.0001; Area under the Curve=0.5779) in the second step. No other variable entered in the model.
